# A central role for PI3K-AKT signaling pathway in linking *SAMHD1*-deficiency to the type I interferon signature

**DOI:** 10.1038/s41598-017-18308-8

**Published:** 2018-01-08

**Authors:** Changhoon Oh, Jeongmin Ryoo, Kiwon Park, Baek Kim, Michele B. Daly, DongYeon Cho, Kwangseog Ahn

**Affiliations:** 1 School of Biological Sciences, Seoul National University, Seoul, 08826 Republic of Korea; 2 Center for RNA Research, Institute for Basic Science (IBS), Seoul, 08826 Republic of Korea; 3 Department of the Interdisciplinary Program in Genetic Engineering, Seoul National University, Seoul, 08826 Republic of Korea; 4 Department of Pediatrics, Emory University School of Medicine, Atlanta, Georgia 30322 USA

## Abstract

The autoimmune disorder Aicardi-Goutières syndrome (AGS) is characterized by a constitutive type I interferon response. SAMHD1 possesses both dNTPase and RNase activities and mutations in *SAMHD1* cause AGS; however, how *SAMHD1*-deficiency causes the type I interferon response in patients with AGS remains unknown. Here, we show that endogenous RNA substrates accumulated in the absence of SAMHD1 act as a major immunogenic source for the type I interferon response. Reconstitution of SAMHD1-negative human cells with wild-type but not RNase-defective SAMHD1 abolishes spontaneous type I interferon induction. We further identify that the PI3K/AKT/IRF3 signaling pathway is essential for the type I interferon response in *SAMHD1*-deficient human monocytic cells. Treatment of PI3K or AKT inhibitors dramatically reduces the type I interferon signatures in *SAMHD1*-deficient cells. Moreover, *SAMHD1*/*AKT1* double knockout relieves the type I interferon signatures to the levels observed for wild-type cells. Identification of AGS-related RNA sensing pathway provides critical insights into the molecular pathogenesis of the type I interferonopathies such as AGS and overlapping autoimmune disorders.

## Introduction

Aicardi-Goutières syndrome (AGS) is a monogenic autoinflammatory disorder that overlaps phenotypically with congenital viral infection and systemic lupus erythematosus (SLE), and is characterized by constitutive upregulation of type I interferon (IFN) in the serum and cerebrospinal fluid^1^. While SLE is associated with more than 20 genes, AGS is caused by autosomal recessive mutations in one of several genes encoding enzymes involved in nucleic acid metabolism (TREX1, RNASEH2, ADAR and SAMHD1) or by gain-of-function mutations in the cytosolic RNA sensor IFIH^12–7^. Some children with AGS also display an early onset form of SLE. Given that the pathology of SLE is complex and heterogeneous, AGS could be an excellent model disease to study systemic autoimmunity and provide a clue to the pathogenesis of SLE.

*SAMHD1* was identified initially as the human ortholog of the mouse IFNγ-induced gene *Mg11* ^8^. Although SAMHD1 has been proposed to serve as a negative regulator of the innate immune response^4^, studies have focused overwhelmingly on the inhibitory mechanism of SAMHD1 against HIV-1 replication because of an unexpected identification of SAMHD1 as an HIV-1 restriction factor^9,10^. In mouse models, mouse SAMHD1 also seems to restrict the replication of certain retroviruses; however, *Samhd1* knockout mice did not display AGS-like symptoms^11,12^. SAMHD1 possesses dual enzymatic activities: deoxynucleoside triphosphohydrolase (dNTPase) and phosphorolytic 3′-5′ exoribonuclease^10,13–16^. The physiological function of SAMHD1 under natural conditions remains poorly understood. In particular, the mechanism by which the mutations in *SAMHD1* cause AGS needs to be determined. Recently, SAMHD1 was implicated in the DNA damage response and in preventing autoimmunity by maintaining genome integrity^17,18^. Considering that all of the AGS-related genes associated with nucleic acid metabolism and nucleic acid sensing dysfunction implicated in autoimmunity^1^, the elevated IFN signature observed in SAMHD1-related AGS patients might be caused by activation of the innate immune response against dysregulated endogenous nucleic acids. In this study, we explored the role of SAMHD1 in regulating nucleic acid-mediated type I IFN signaling to understand the molecular pathogenesis of AGS and overlapping autoimmune disorders.

## Results

### *SAMHD1*-deficient human monocytic cells display a heightened IFN signature

**Figure 1:**
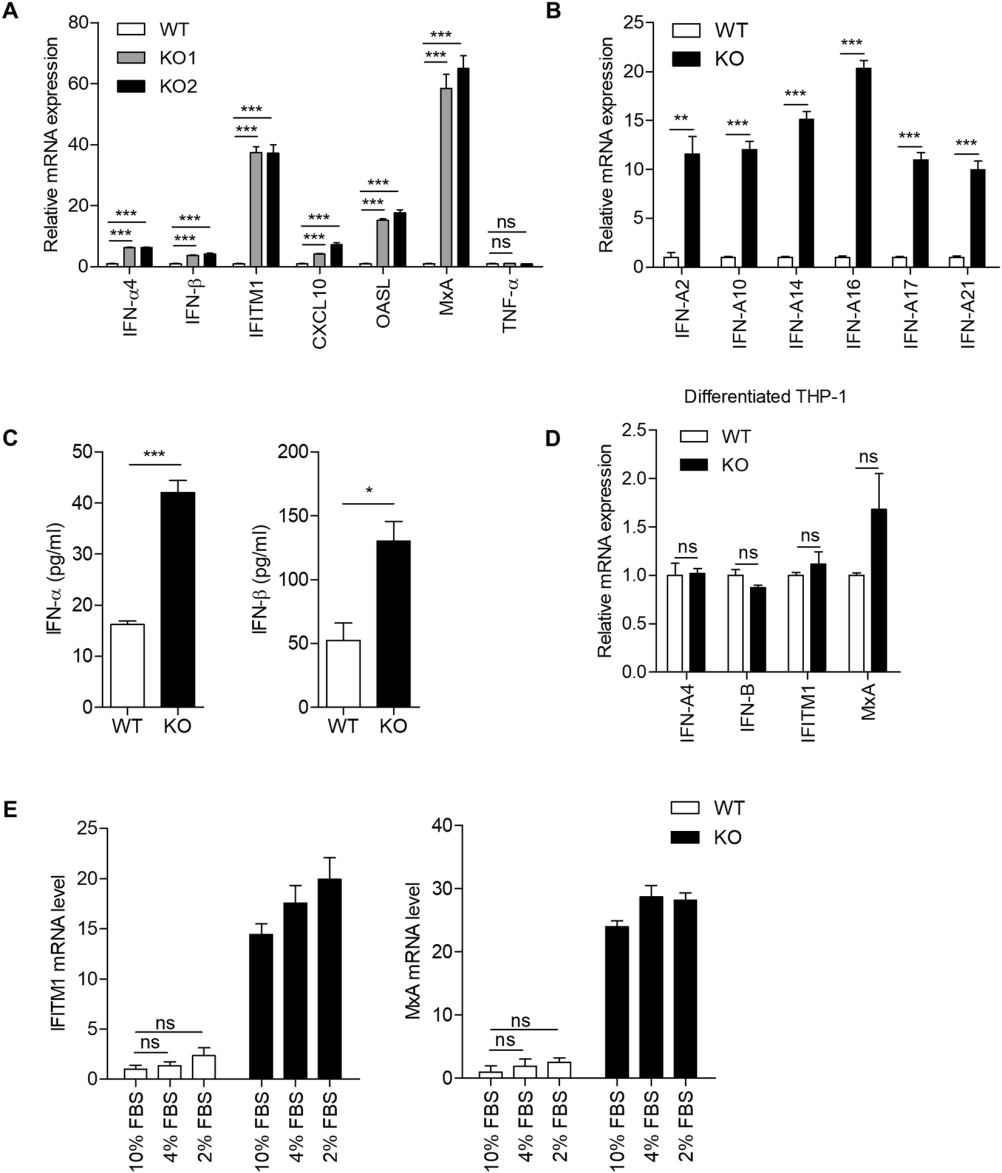
Knockout of *SAMHD1* activates an immune response in human monocytic cells. (**A**,**B**) Relative mRNA levels for the indicated genes in *SAMHD1*-deficient cells, as assessed by qRT-PCR and normalized to *β-actin* expression. (**C**) ELISA of IFN-α production in cell extracts. ELISA of IFN-β production in supernatants. Conditioned media were concentrated using Amicon Ultra-15 before analysis. (**D**) qRT-PCR analysis of *IFN-α*, *IFN-β*, *IFITM1* and *MxA* in PMA-differentiated wild-type and *SAMHD1*-deficient THP-1 cells. (**E**) Wild-type and *SAMHD1*-deficient THP-1 cells were incubated in basal media containing 10% FBS or reduced-serum media containing 4% or 2% FBS for 48 h, followed by qRT-PCR analysis of *IFITM1* and *MxA* mRNA levels. Data were standardized to *β-actin*. In (**A**–**E**), data represent the mean ± SEM of triplicate independent experiments (*p ≤ 0.05, **p ≤ 0.01, ***p ≤ 0.001, ns: not significant, two-tailed Student’s t-test).

AGS is a type of systemic inflammatory disease that has a type I IFN signature in human. However, *Samhd1*-deficient mice displayed neither significant upregulation of IFN-stimulated genes (ISGs) in their sera nor other systemic autoimmune phenotypes, although *Samhd1* ^−/−^ mouse cells exhibited spontaneous induction of ISGs^11,12^. Therefore, we used human THP-1 cells, a well-established model of human monocytes, to study the molecular pathology of the SAMHD1-related AGS. We analyzed transcriptional signature of AGS using two independent *SAMHD1*-deficient THP-1 cells engineered by different clustered regularly interspaced short palindromic repeats (CRISPR) single guide RNAs (sgRNAs). Type I interferons including various subtypes of *IFN-α* and *IFN-β*, and ISGs (*IFITM1*, *CXCL10*, *OASL*, and *MxA*) were upregulated in *SAMHD1*-deficient cells (Fig. 1A and B), which was largely consistent with the results obtained from the samples of patients with AGS^19^. Notably, the level of tumor necrosis factor alpha (TNF-α) was similar between *SAMHD1*-deficient and wild-type cells (Fig. 1A), suggesting that SAMHD1 is not involved in the nuclear factor-kappa B (NF-κB) pathway. The transcriptional induction of type I IFNs resulted in increased synthesis of type I IFN proteins in *SAMHD1*-deficient cells, as assessed by enzyme-linked immunosorbent assays (ELISAs) (Fig. 1C). To investigate the effect of the differentiation of THP-1 cells on the type I IFN signaling in *SAMHD1*-deficiency, we compared the expressions of type I IFN and ISGs in PMA-differentiated *SAMHD1*-deficient cells to those in PMA-differentiated wild-type cells (Fig. 1D). Interestingly, differentiated THP-1 cells did not show a distinct IFN signature in spite of *SAMHD1*-deficiency, proposing that SAMHD1 related type I IFN response would be differentially displayed in cell type specific manner. Primary fibroblasts from SAMHD1-related AGS patients exhibited reduced proliferation and a delay in cell cycle progression^17^. In agreement with this observation, *SAMHD1*-deficient THP-1 cells also displayed delayed cell cycle progression (Supplementary Figure S1). To assess the relation of type I IFN response with a delayed cell cycle progression in *SAMHD1*-deficient cells, we regulated the cell cycle progression by alternating the serum concentration in culture media^20^ (Supplementary Figure S2). Low serum conditioning could not induce a distinct IFN signature in both wild-type and *SAMHD1*-deficient cells (Fig. 1E), suggesting that cell cycle delay do not explain the autoimmune phenotypes in *SAMHD1*-deficient cells.

**Figure 2:**
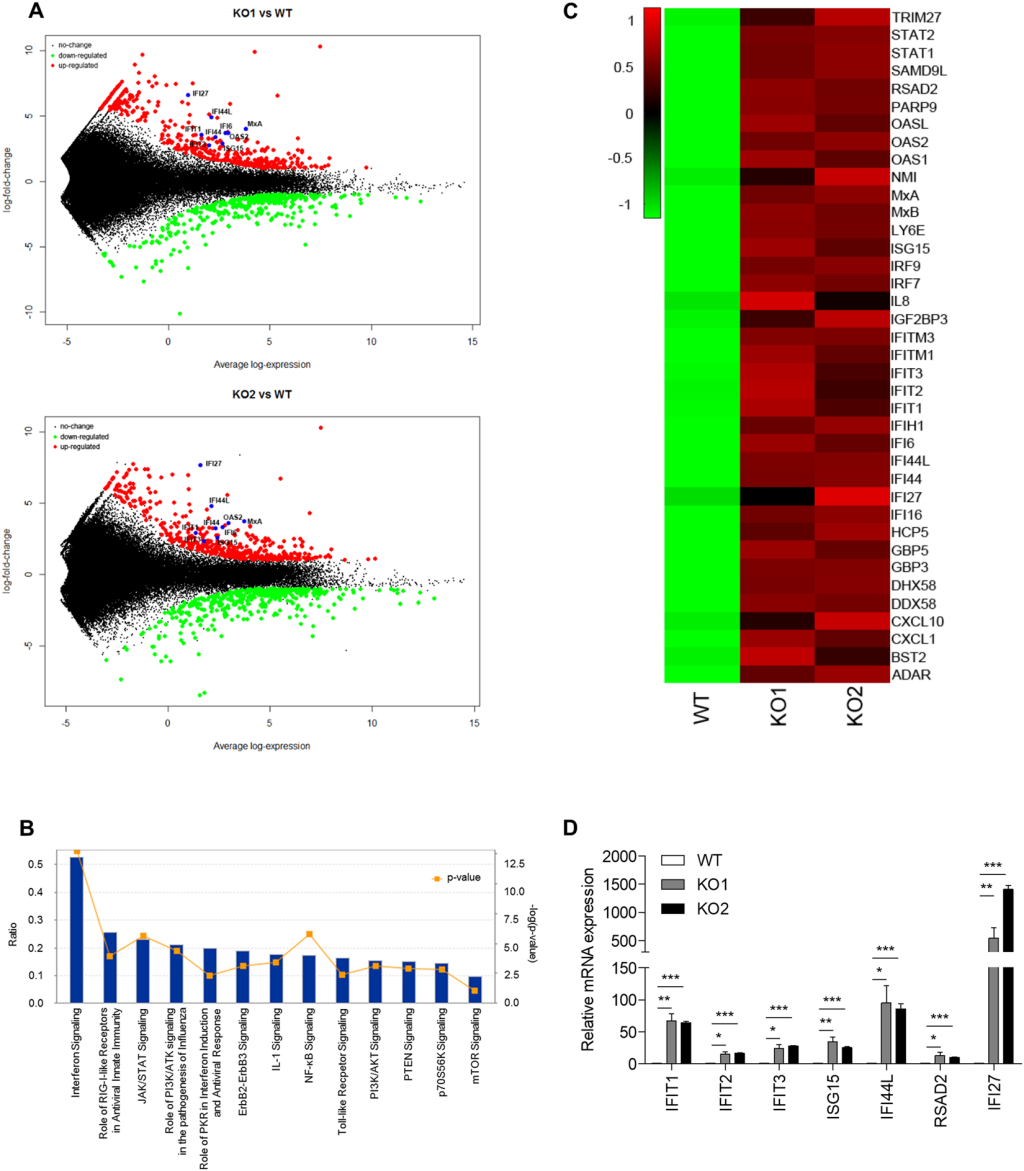
Identification and classification of DEGs in *SAMHD1*-deficient THP-1 cells. (**A**) RNA-seq MAplot of wild-type versus *SAMHD1* knockout cells as indicated. Three biological replicates were analyzed for both data sets. Average gene expression is plotted on the x-axis and log2 fold-change is plotted on the y-axis; red dots: upregulated genes (log2 FC ≥ 1 and adjusted p-values < 0.01), green dots: downregulated genes (log2 FC ≤ −1 and adjusted p-values < 0.01), blue dots: ISGs. (**B**) Statistically significant signaling pathways for genes upregulated by over 2-fold in *SAMHD1* knockout samples were obtained by Ingenuity Pathway Analysis (IPA). Blue bars indicate the ratio of the total number of genes involved in the specific pathway versus input list genes, while the orange squares show −log (p-value). (**C**) Heatmap of ISGs expressed in the indicated cells with the RNA-seq data. Gene expression levels (averaged reads per kilobase per million mapped reads (RPKM) values over 3 replicates) was standardized and clustered based on the dissimilarity values (1-Pearson correlation) between genes using the average linkage method as shown in the dendrogram. (**D**) The mRNA levels of ISGs in wild-type and *SAMHD1*-deficient THP-1 cells were determined by qRT-PCR. Data were normalized to the *GAPDH* level. Data represent the mean ± SEM of triplicate independent experiments (*p ≤ 0.05, **p ≤ 0.01, ***p ≤ 0.001, two-tailed Student’s t-test).

We then performed RNA sequencing (RNA-seq) to ascertain the IFN signatures of AGS. *SAMHD1*-deficient cells showed distinct mRNA expression patterns among more than 1200 genes (Fig. 2A). Pathway enrichment analysis revealed that the upregulated genes were significantly associated with the immune system, IFN signaling and cytokine signaling pathways (Fig. 2B). Upstream regulator analysis predicted that various cytokines and transcription regulators related to the induction of type I IFN and ISGs would be activated (Supplementary Figure S3). In particular, a broad range of ISGs were upregulated in *SAMHD1*-deficient cells (Fig. 2C), which closely resembled the IFN signature observed for patients with SLE and AGS^19^. The expressions of several ISGs were validated by quantitative real-time reverse transcription polymerase chain reaction (qRT-PCR) (Fig. 2D). These results indicated that *SAMHD1*-deficient THP-1 cells show spontaneous autoimmune phenotypes.

### RNA enriched in the absence of SAMHD1 is a major source of the IFN-α response

**Figure 3:**
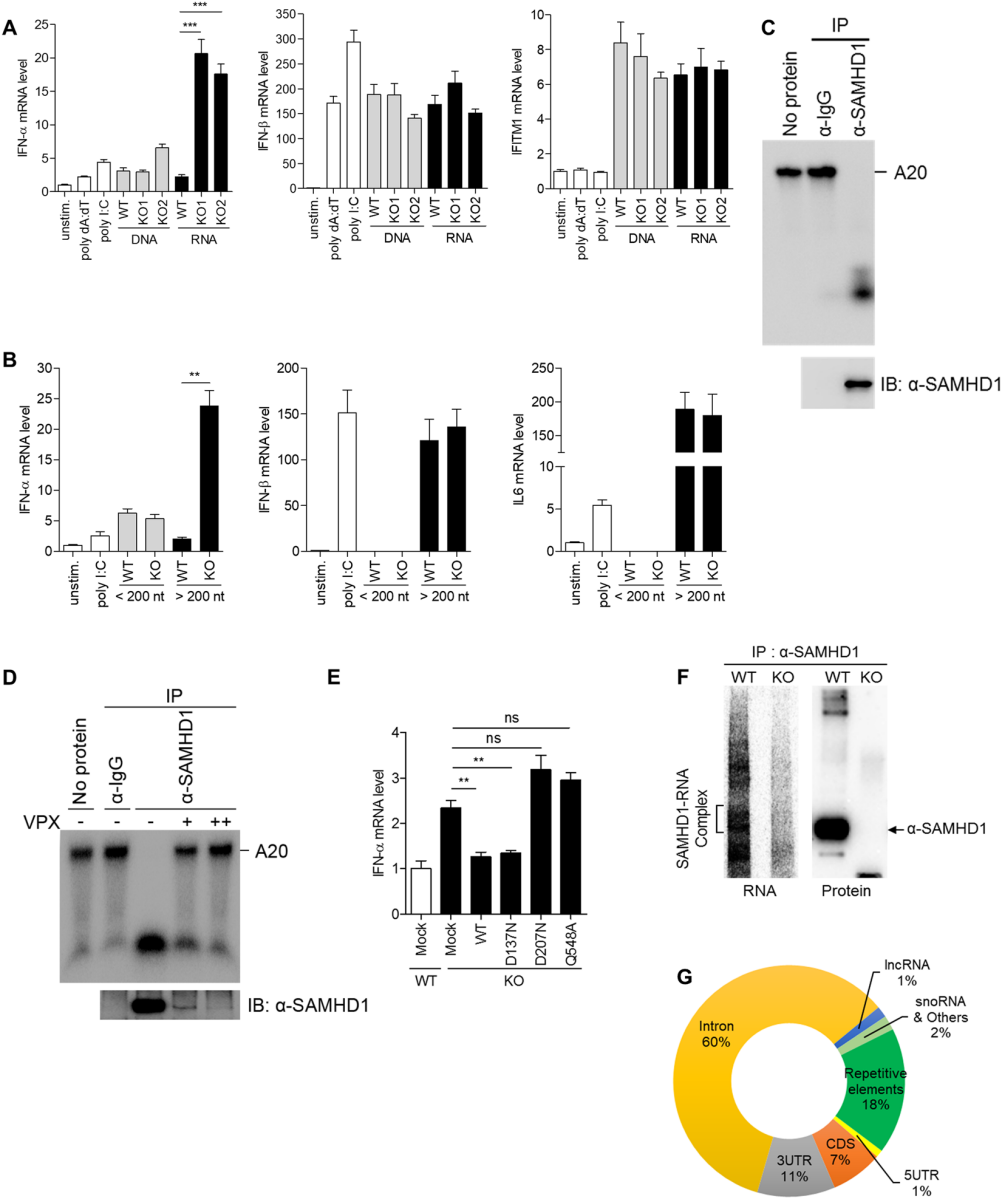
Accumulated RNAs in *SAMHD1*-deficient cells function as immune stimuli. (**A**,**B**) PMA-differentiated wild-type THP-1 cells were stimulated with poly dA:dT, poly I:C, an equal amount (5 μg/ml) of isolated total DNA and RNA from wild-type and *SAMHD1*-deficient cells, or left unstimulated (**A**). Total RNA isolated from wild-type and *SAMHD1*-deficient cells were further size-fractionated and an equal amount of RNA from each fraction was used to stimulate PMA-differentiated wild-type THP-1 cells (**B**), followed by qRT-PCR analysis of *IFN-α*, *IFN-β*, *IFITM1* and *IL6* mRNA levels. (**C**,**D**) *In vitro* RNase activity assay for SAMHD1 immunopurified from undifferentiated THP-1 cells using A20 single-stranded RNA substrates. An isotype-matched control anti-IgG and anti-SAMHD1 antibodies were used for immunopurification. THP1 cells were infected with serial dilution of Vpx-loaded or control SIV VLPs (**D**). (**E**) qRT-PCR analysis of *IFN-α* in wild-type and *SAMHD1*-deficient cells reconstituted with indicated SAMHD1 wild-type and mutant constructs. (**F**) Autoradiography of SAMHD1-RNA complex and western blotting of SAMHD1 protein immunoprecipitated from SAMHD1 CLIP. (**G**) Pie chart showing the distribution of statistically significant peaks (q < 0.001) among the indicated RNA classes. Data were normalized to *β-actin* expression. In (**A**), (**B**) and (**E**), these data represent the mean ± SEM of triplicate independent experiments (**p ≤ 0.01, ***p ≤ 0.001, ns: not significant, two-tailed Student’s t-test).

We examined whether inappropriate accumulation of nucleic acids in *SAMHD1*-deficient cells activates IFN responses. We isolated both DNA and RNA from wild-type and *SAMHD1*-deficient cells and examined their abilities to stimulate the IFN response in phorbol 12-myristate 13-acetate (PMA)-differentiated THP-1 cells. Interestingly, only RNA derived from *SAMHD1*-deficient cells, but not that from wild-type cells, highly activated *IFN-α* expression. On the other hand, DNA isolated from wild-type and *SAMHD1*-deficient cells had comparable abilities to activate *IFN-α* expression (Fig. 3A, left). RNA purified from *SAMHD1*-deficient cells had no discernible impact on the expression of *IFN-β* and *IFITM1* mRNA compared with RNA from wild-type cells (Fig. 3A, middle and right). Considering that the isolated RNAs are likely composed of various RNA species, the *IFN-β* induction by RNA species that activate retinoic acid-inducible gene-I (RIG-I) or melanoma differentiation associated gene 5 (MDA5)-dependent pathways could mask the *IFN-β* induction by RNA substrates of SAMHD1. To investigate the detailed features of IFN-stimulatory RNA species, we isolated small (<200 nt) and large (>200 nt) RNAs in two separate fractions from each of the wild-type and *SAMHD1*-deficient THP-1 cell lines and then stimulated PMA-differentiated THP-1 cells with these RNAs. The large RNAs (>200 nt) from *SAMHD1*-deficient cells activated *IFN-α* mRNA expression significantly (Fig. 3B, left) and showed similar effects on the induction of *IFN-β* or *IL-6* mRNA compared with the large RNAs (>200 nt) from wild-type cells (Fig. 3B, middle and right). Cell fractionation experiments also revealed that the cytoplasmic, but not the nuclear, RNA of *SAMHD1*-deficient cells induced *IFN-α* expression dominantly (Supplementary Figure S4). Our data suggested that the cytoplasmic RNA (>200 nt) accumulated in the absence of SAMHD1 triggers the IFN response. To investigate the discrepancy of type I IFN response in the absence of SAMHD1 between undifferentiated and differentiated THP-1 cells, we repeated the experiment for RNA stimulation with RNA isolated from differentiated wild-type and *SAMHD1*-deficient THP-1 cells. These RNAs did not show significant differences in activating type I IFN response (Supplementary Figure S5A), suggesting that the expression and accumulation of RNA substrates of SAMHD1 are differentially regulated after differentiation of monocytes^21,22^ and these differences cause the cell type specificity of SAMHD1 related autoimmune response. Furthermore, we performed the RNA stimulation with RNA purified from undifferentiated wild-type and *SAMHD1*-deficient cells into undifferentiated wild-type or *SAMHD1*-deficient cells. Consistent with the data with differentiated THP-1 cells, RNA derived from *SAMHD1*-deficient cells distinctly activated *IFN-α* expression (Supplementary Figures S5B and S5C). The activation of IFN response in undifferentiated cells was relatively mild compared to that of differentiated THP-1 cells. However, RNAs isolated from *SAMHD1*-deficient cells activated IFN-α expression about 3-fold higher than the RNAs isolated from wild-type cells in both undifferentiated wild-type and *SAMHD1*-deficient THP-1 cells. Thus, it seems that SAMHD1 could not efficiently degrade the transfected RNA, even though SAMHD1 degrades endogenous RNA species via its RNase activity.

SAMHD1 possesses both dNTPase and RNase activity, both of which are relevant to nucleic acid metabolism. We examined which of the two SAMHD1 functions is involved in the regulation of the type I IFN response. The RNase activity of SAMHD1 in undifferentiated THP-1 cells has not been characterized; therefore, we first examined whether the SAMHD1 protein immunopurified from undifferentiated THP-1 cells possesses RNase activity. Immunopurified SAMHD1 protein was able to digest single-stranded RNA efficiently (Fig. 3C). To exclude the possibility that contaminated RNA exonuclease during SAMHD1 purification might be responsible for the observed RNase activity, we added Vpx-mediated SAMHD1 depleted controls (Fig. 3D). Vpx-mediated degradation of SAMHD1 significantly reduced the enzymatic activity of immunopurified SAMHD1 protein, suggesting that SAMHD1 possesses RNase activity in undifferentiated THP-1 cells. Previously, we identified *SAMHD1* point mutations that caused loss of one or both functions; RNase^−^/dNTPase^−^ SAMHD1_D207N_, RNase^+^/dNTPase^−^ SAMHD1_D137N_ and RNase^−^/dNTPase^+^ SAMHD1_Q548A_^15^. We reconstituted *SAMHD1*-deficient THP-1 cells with these SAMHD1 mutants using transient transfection and monitored the expression of *IFN-α* mRNA. SAMHD1_D207N_ and SAMHD1_Q548A_, which have no RNase activity, did not repress the *IFN-α* induction. Notably, wild-type SAMHD1 and SAMHD1_D137N_ reduced the expression of *IFN-α* to the level observed in the mock control cells (Fig. 3E). These data supported the view that in *SAMHD1*-deficient cells, accumulation of incompletely digested RNAs leads to activation of IFN immune response.

To gain the insight into the nature of cellular immune stimulatory RNA, we performed high-throughput sequencing of RNA isolated by crosslinking immunoprecipitation (HITS-CLIP) analysis with SAMHD1. Immunoprecipitates of SAMHD1 protein and SAMHD1-RNA complex were successfully detected by western blot and autoradiography (Fig. 3F). A total of 5,500,000 peaks covering 38,000 genes including endogenous retroelements were called through SAMHD1 CLIP-seq. 60% of these peaks were mapped to intronic region, suggesting that SAMHD1 would function in nucleus during RNA processing or regulate RNA debris originated from intron in cytoplasm. 11% of peaks were mapped to 3′ UTR and only 8% of peaks were mapped to 5′ UTR and CDS region (Fig. 3G). Notably, 18% were repetitive elements that have been suggested as the source of endogenous immune stimulatory nucleic acid to account for AGS. Our data showed that significant portion of the retroelement RNAs that are identified in SAMHD1 CLIP-seq were upregulated in *SAMHD1*-deficient cells. We, thus, speculate that these retroelement RNAs could be the substrates of SAMHD1 and function as immune stimulatory RNAs in *SAMHD1*-deficient cells.

### ISG activation in *SAMHD1*-deficient cells is dependent on IRF3 & Type I IFN receptor

**Figure 4:**
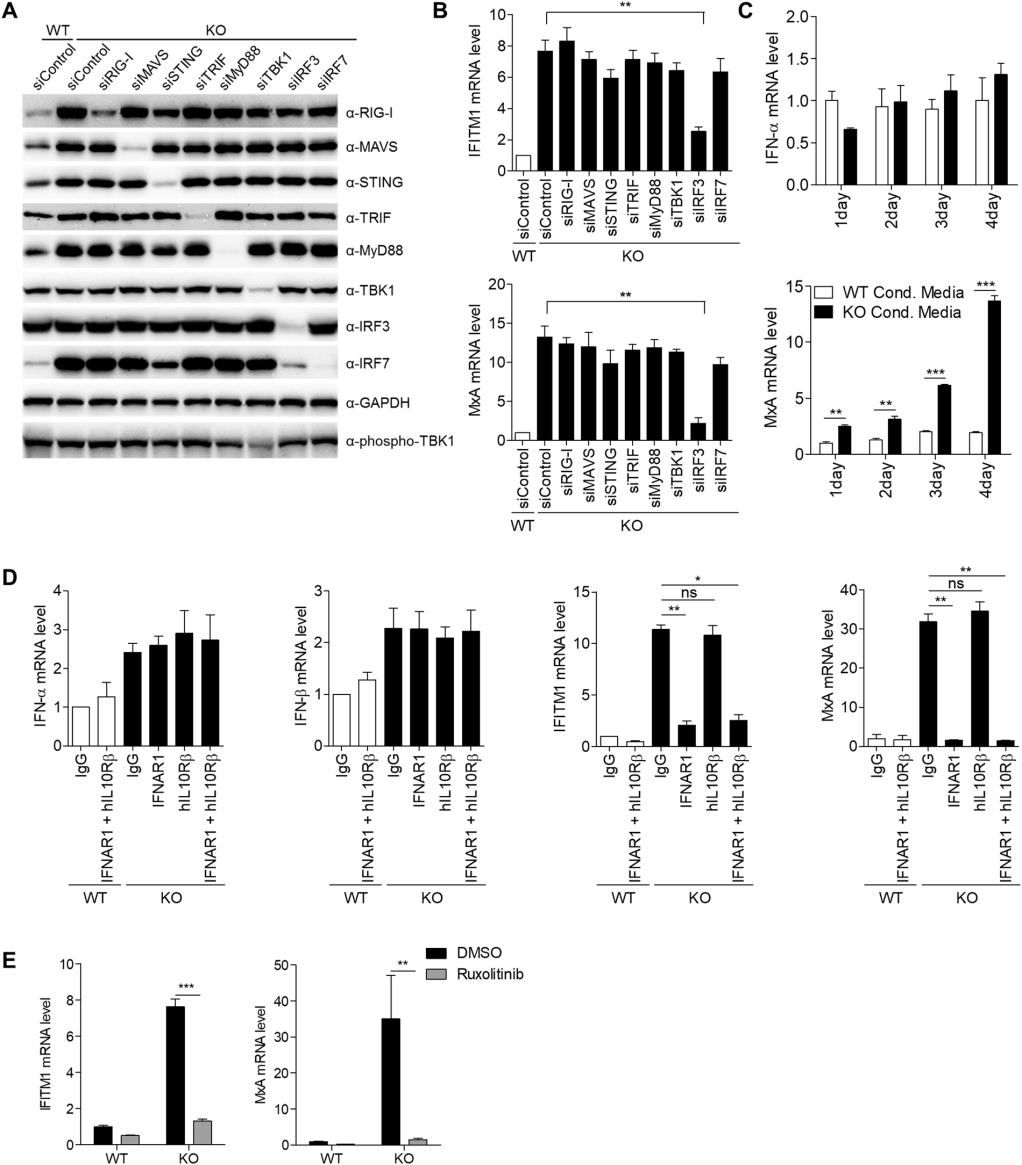
ISGs induction in *SAMHD1*-deficiency occurs through IRF3 and type I IFN receptor signaling pathway. (**A**,**B**) Wild-type and *SAMHD1*-deficient THP-1 cells were transfected with control siRNA or specific siRNA for the indicated genes for 72 h. Cell lysates were subjected to western blotting to analyze the protein levels. GAPDH was loaded as a control (**A**). The cellular mRNA was analyzed by qRT-PCR to determine the expression of *IFITM1* and *MxA* relative to *GAPDH* (**B**). (**C**) qRT-PCR analysis of *IFN-α* and *MxA* in THP-1 cells incubated with conditioned media (Cond. Media.) from wild-type or *SAMHD1*-deficient cells for the indicated days. (**D**) qRT-PCR analysis of *IFN-α*, *IFN-β*, *IFITM1* and *MxA* in wild-type and *SAMHD1*-deficient THP-1 cells treated with control IgG or neutralizing antibodies against type I and III IFN receptor subunits as indicated. Results were standardized to *β-actin* levels. (**E**) The measurement of *IFITM1* and *MxA* mRNA expressions in *SAMHD1*-deficient cells by qRT-PCR after treating *SAMHD1*-deficient cells with control dimethylsulfoxide (DMSO) or 2 µM Ruxolitinib for 24 h. Data were standardized to *GAPDH*. In (**B**–**E**), these data represent the mean ± SEM of triplicate independent experiments. (*p ≤ 0.05, **p ≤ 0.01, ***p ≤ 0.001, ns: not significant, two-tailed Student’s t-test).

We sought to identify the RNA sensing pathway associated with ISG induction in *SAMHD1*-deficient cells. RIG-I-like receptors (RLRs), which include RIG-I and MDA5, and intracellular Toll-like receptors (TLRs) are well known cytosolic RNA sensors^23–26^. We assumed that one of these RNA sensors might be linked to the IFN signature in *SAMHD1*-deficient cells; therefore, depleted each of these sensors or downstream molecules independently using short interfering RNA (siRNA) treatment (Fig. 4A and Supplementary Figure S6A). All of the known RNA sensors and intermediate signaling molecules, except Interferon Regulatory Factor 3 (IRF3), were dispensable for the spontaneous IFN signature in *SAMHD1*-deficient cells (Fig. 4B and Supplementary Figure S6B). IRF3 depletion in *SAMHD1*-deficient cells abrogated the expression of *IFITM1* and *MxA* to near the levels of wild-type THP-1 cells. Activation of almost known RNA sensing pathways requires the phosphorylation of TBK1, which acts upstream of IRF3. Notably, phosphorylated TBK1 levels were similar between wild-type and *SAMHD1*-deficient cells (Fig. 4A), suggesting that a novel RNA sensing pathway is responsible for the IFN response observed in *SAMHD1*-deficient cells.

To investigate whether the proteins secreted from *SAMHD1*-deficient cells boost ISG induction, we harvested the conditioned media from wild-type and *SAMHD1*-deficient THP-1 cells. The conditioned media from both wild-type and *SAMHD1*-deficient cells did not exhibit any significant differential ability to stimulate *IFN-α* expression. However, wild-type THP-1 cells treated with the conditioned media from *SAMHD1*-deficient cells displayed significantly elevated ISG levels in a time-dependent manner (Fig. 4C). Both type I (IFN-α and IFN-β) and type III (IFN-λ) IFNs can lead to the similar spectrum of ISG induction. To identify the discrete contribution of type I and type III IFNs to ISG induction, *SAMHD1*-deficient cells were treated with neutralizing antibodies directed against the type I and III IFN receptor subunits, IFNAR1 and IL10Rβ, respectively. Neither the anti-IFNAR1 nor anti-IL10Rβ antibodies reduced the expression of type I IFN in *SAMHD1*-deficient cells. However, the expressions of ISGs (*IFITM1* and *MxA*) were reduced to basal levels in cells treated with anti-IFNAR1, but not in those treated with the anti-IL10Rβ antibody. Co-treatment with both IFNAR1 and IL10Rβ antibodies did not show synergistic inhibitory effects on ISGs induction (Fig. 4D). Thus, we concluded that type I IFN is responsible for the IFN signature observed in *SAMHD1*-deficient cells. Type I IFN receptors associate with Janus activated kinases (JAKs), and activation of the Janus kinase/signal transducers and activators of transcription (JAK/STAT) pathway is indispensable for ISGs induction^27^. JAK inhibitor treatment diminished ISG induction thoroughly (Fig. 4E). Taken together, these findings indicated that the type I IFN secreted by the IRF3-dependent novel RNA sensing pathway activates ISG induction, which could be responsible for AGS pathogenesis.

### The PI3K/AKT signaling pathway is involved in linking *SAMHD1*-deficiency to the IFN response

**Figure 5:**
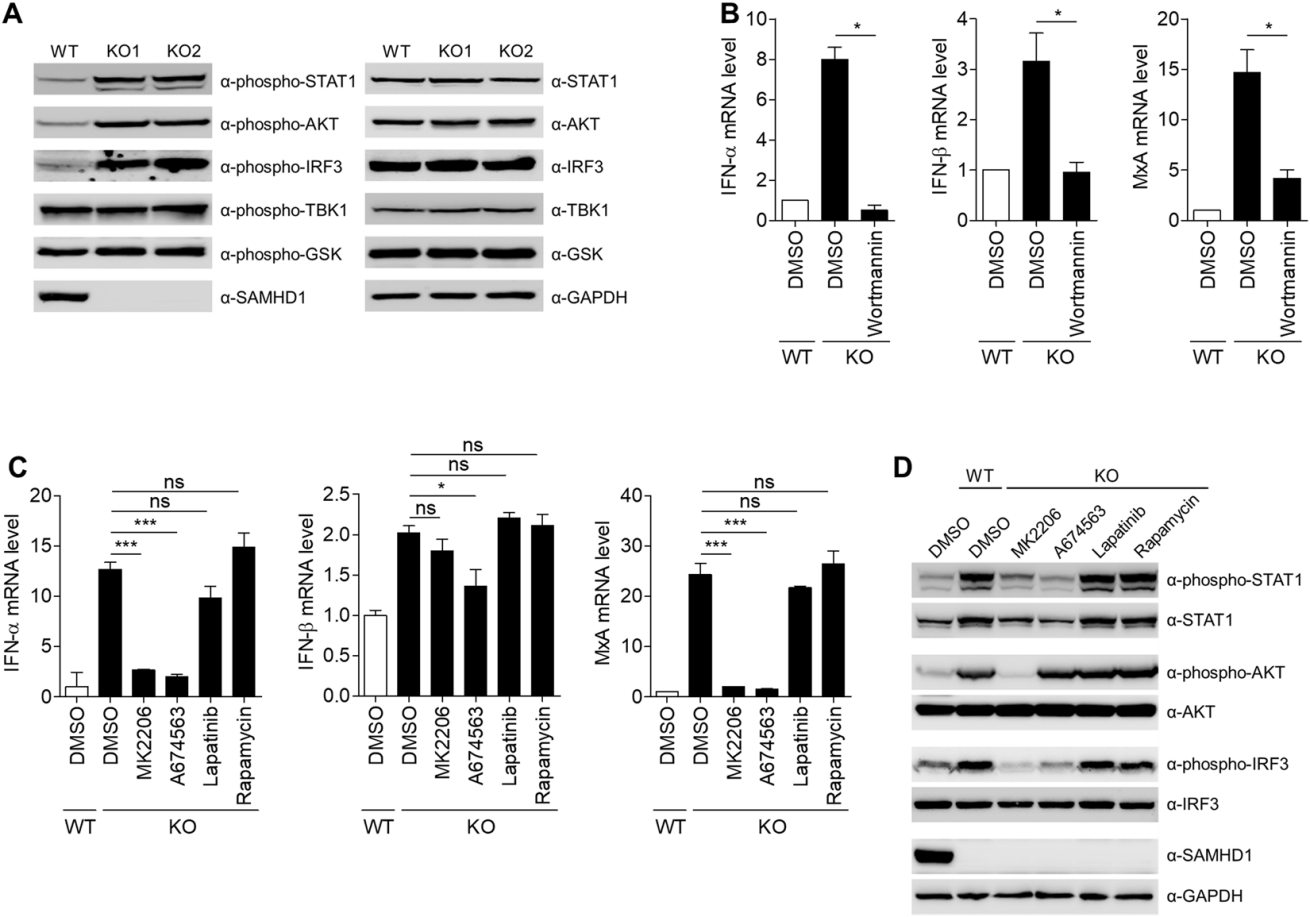
The PI3K/AKT pathway is responsible for linking *SAMHD1*-deficiency to the IFN response. (**A**) Western blotting analysis of cell extracts from wild-type and two independent *SAMHD1*-deficient cell lines using the indicated antibodies. (**B**) qRT-PCR analysis of *IFN-α*, *IFN-β* and *MxA* levels in wild-type and *SAMHD1*-deficient THP-1 cells treated with control DMSO or 1 µM Wortmannin for 24 h. Data were normalized to *GAPDH* levels. (**C**,**D**) Wild-type and *SAMHD1*-deficient THP-1 cells were treated with control DMSO, 1 µM MK2206, 1 µM A674563, 0.1 µM Lapatinib or 50 nM Rapamycin for 24 h. Cells were analyzed for the mRNA levels of *IFN-α*, *IFN-β* and *MxA* relative to *GAPDH* by qRT-PCR (**C**) and for the western blotting analysis to determine the phosphorylation status of STAT1, AKT and IRF3 (**D**). GAPDH served as a loading control. In (**B**) and (**C**), these data represent the mean ± SEM of triplicate independent experiments (*p ≤ 0.05, ***p ≤ 0.001, ns: not significant, two-tailed Student’s t-test).

**Figure 6:**
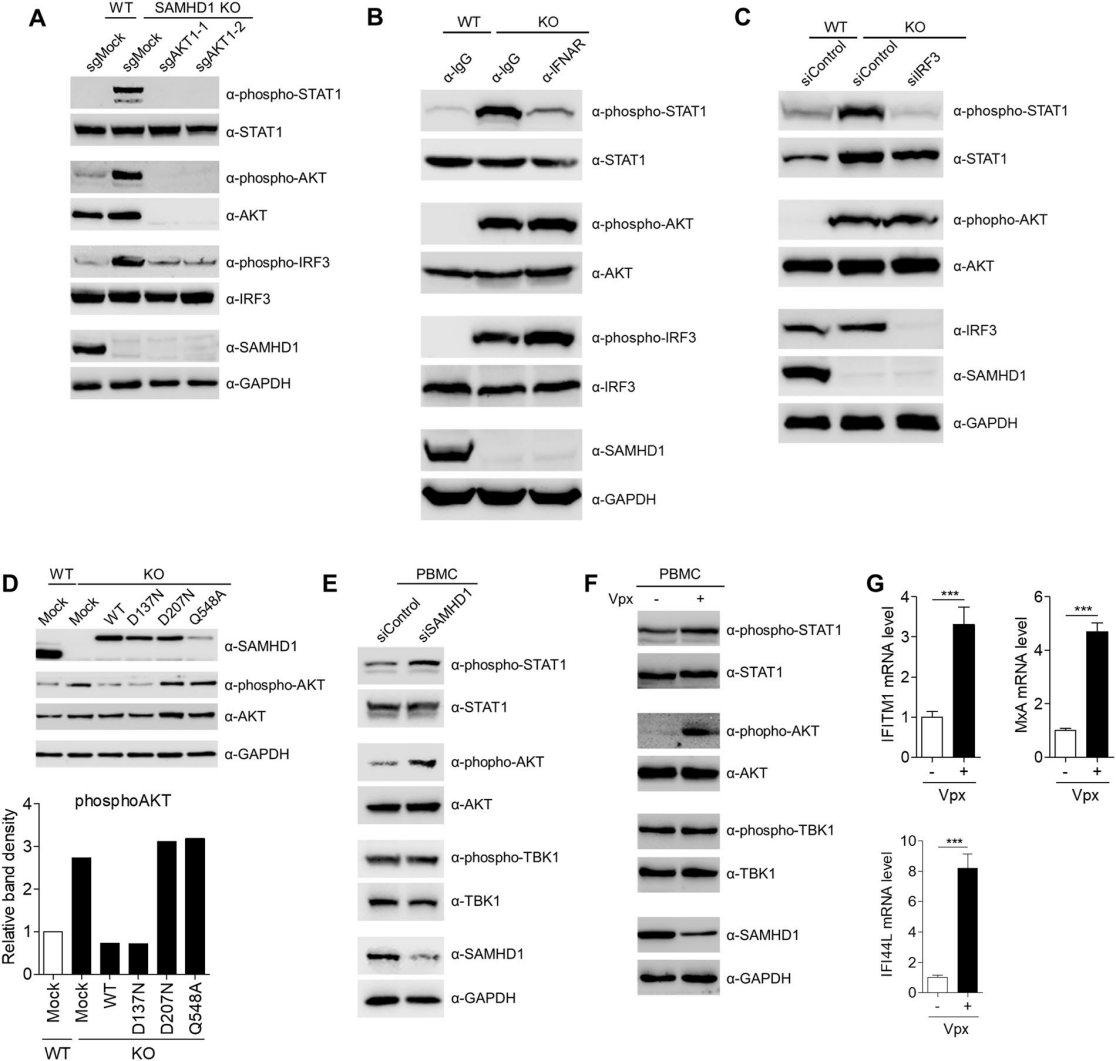
The PI3K/AKT functions upstream of IRF3 to activate type I IFN response. (**A**) The phosphorylation status of STAT1 and IRF3 in wild-type, *SAMHD1* knockout and *SAMHD1*/*AKT* double-knockout THP-1 cells was determined by western blotting analysis. (**B**,**C**) Western blotting analysis of cell extracts from wild-type and *SAMHD1*-deficient cells treated with control IgG or a neutralizing antibody against a type I IFN receptor subunit for 48 h (**B**) or transfected with control non-specific siRNA or a specific siRNA for IRF3 (**C**). (**D**) Determination of the phosphorylation status of AKT after reconstitution of *SAMHD1*-deficient cells with wild-type and SAMHD1 mutants by western blotting analysis. Graph shown below indicates the ratio of phosphoAKT to wild-type control. (**E**) Isolated PBMCs from four donors were transfected with SAMHD1-specific siRNA or control non-specific siRNA for two cycles to enhance the knockdown efficiency. After 48 h of incubation, PBMCs were analyzed by western blotting to monitor the activation of STAT1 and AKT. Data are representative of four independent experiments with similar results. (**F**,**G**) PBMCs were infected with Vpx-loaded or control SIV VLPs. After 96 h of incubation, cells were analyzed for the western blotting analysis to determine the phosphorylation status of STAT1 and AKT (**F**) and for the mRNA levels of *IFITM1*, *MxA* and *IFI44L* relative to *GAPDH* by qRT-PCR (**G**). In (**A**–**F**), GAPDH served as a loading control. In (**G**), data represent the mean ± SEM of triplicate independent experiments (***p ≤ 0.001, two-tailed Student’s t-test).

Type I IFN can activate multiple signaling pathways, including the JAK/STAT pathway, the p38 and extracellular signal-regulated kinase (ERK) pathways, and the phosphoinositide 3-kinase (PI3K)/AKT pathway^28^. Pathway enrichment analysis of genes upregulated in *SAMHD1*-deficient cells showed a significant enrichment of genes related to the PI3K/AKT pathway (Fig. 2B). Indeed, STAT1 and AKT were highly activated in *SAMHD1*-deficient cells, as assessed by the phosphorylation levels of these molecules (Fig. 5A). IRF3 was also activated in *SAMHD1*-deficient cells, which agreed with the observation that depletion of IRF3 abrogates ISG induction (Fig. 4B). However, p38 and ERK pathways were not activated in *SAMHD1*-deficient cells (Supplementary Figure S7A). To ascertain the role of the PI3K/AKT pathway in the SAMHD1-related IFN response, we treated *SAMHD1*-deficient cells with wortmannin, a PI3K inhibitor. Wortmannin inhibited not only the induction of type I IFN and ISGs but also the activation of AKT and STAT1 (Fig. 5B and Supplementary Figure S7B). Treatment of *SAMHD1*-deficient cells with the pan-AKT inhibitor MK2206 or the AKT1 inhibitor A674563 reduced the IFN signature. By contrast, inhibition of the PI3K upstream tyrosine kinase using Lapatinib and inhibition of mTOR, a downstream effector of AKT, using rapamycin, did not affect the IFN signature (Fig. 5C and Supplementary Figure S7C). Consistent with these data, the activation of STAT1 is also abrogated by AKT inhibitors treatment (Fig. 5D). To confirm the data obtained from the pharmacological studies, we generated the *SAMHD1*/*AKT1* double knockout cells via the CRISPR/ CRISPR associated protein9 (Cas9) system. Phosphorylation of STAT1 and IRF3 induced by *SAMHD1* knockout was markedly diminished upon *SAMHD1*/*AKT1* double knockout (Fig. 6A). Type I IFN production and ISGs induction also significantly decreased in *SAMHD1*/*AKT1* double knockout cells (Supplementary Figure S8). Glycogen synthase kinase 3-β (GSK3-β) regulates TLR4 mediated IFN-β production negatively^29^. The phosphorylation levels of GSK3-β were similar in the wild-type and *SAMHD1*-deficient cells (Fig. 5A); therefore, the SAMHD1-related type I IFN response is likely independent of the GSK3-β pathway. The PI3K/AKT pathway can be also activated by type I IFN; thus, we attempted to clarify the cause-effect relationship between type I IFN production and activation of the PI3K/AKT pathway. First, we investigated whether treatment of AKT inhibitor abolishes the activation of IRF3. As reported, MK2206 inhibited AKT1 phosphorylation, while A674563, which blocks the phosphorylation of AKT downstream targets, does not. Both AKT inhibitors abrogate IRF3 phosphorylation (Fig. 5D). Neutralizing antibodies against the type I IFN receptor inhibited STAT1 phosphorylation, but not that of AKT or IRF3 (Fig. 6B). siRNA-mediated depletion of IRF3 diminished STAT1 phosphorylation, but not that of AKT (Fig. 6C), suggesting that the PI3K/AKT pathway functions upstream of IRF3 activation in the type I IFN-producing pathway. Taken together, these results showed that IFN production in *SAMHD1*-deficient cells occurs through the PI3K/AKT/IRF3 signaling axis.

The phosphorylation of AKT induced by *SAMHD1* knockout was antagonized by ectopic expression of wild-type SAMHD1 or RNase^+^/dNTPase^−^ SAMHD1_D137N_. Expression of the RNase-defective mutants, SAMHD1_D207N_ and SAMHD1_Q548A_, had little effect on AKT phosphorylation (Fig. 6D), which was consistent with the observation that the RNase activity of SAMHD1 is critical to suppress the spontaneous IFN response in *SAMHD1*-deficient cells (Fig. 3E). To explore the physiological relevance of our findings, we analyzed the role for SAMHD1 in regulating an IFN response using human peripheral blood mononuclear cells (PBMCs). In human PBMCs, not only siRNA-mediated SAMHD1 silencing (Fig. 6E) but also Vpx-mediated degradation of SAMHD1 (Fig. 6F,G) elicited the activation of STAT1 and AKT and the induction of ISGs, but had no effect on TBK1 activation, recapitulating the phenotypes seen in *SAMHD1*-deficient THP-1 cells. By comparison, knockout of *SAMHD1* in HEK293T and HeLa cells did not result in activation of STAT1 and AKT or the induction of ISGs (Supplementary Figure S9), which suggested that the type I interferonopathy associated with *SAMHD1*-deficiency is cell type-specific.

## Discussion

SAMHD1 had been identified as HIV-1 restriction factor in the last decade and it was initially reported to inhibit HIV-1 replication through its dNTPase catalytic activity^30,31^. Later we reported that SAMHD1 restricts HIV-1 and retroviruses through its RNase activity^15,32^. However, several studies reported that they have not observed the RNase activity of SAMHD1, even though SAMHD1 could bind nucleic acids such as ssRNA^33,34^. To clarify this discrepancy, we recently showed that SAMHD1 is a phophorolytic exoribonuclease with A or U base preference^16^. Nevertheless, the existence of RNase activity of SAMHD1 is still being debated.

The current study identified the signaling mechanism driving the chronic upregulation of type I IFN in AGS relating to *SAMHD1*-deficiency. Our findings showed that the RNA species larger than 200 nucleotides that accumulated in *SAMHD1*-deficient cells trigger a type I IFN response. More specifically, we observed that cytosolic RNA species larger than 200 nucleotides are responsible for the type I IFN induction. This type I IFN response does not involve any of the known signaling pathways converging on the activation of TBK1. Instead, the type I IFN response occurs via a novel PI3K/AKT/IRF3 signaling pathway. Consequently, ISGs are induced in bystander cells via a type I IFN receptor- and JAK/STAT-dependent manner (Supplementary Figure S10).

In contrast to AGS patients with mutations in *SAMHD1* ^4^, *Samhd1*-deficient mice did not exhibit any distinct clinical phenotypes^11,12^. The discrepancy between the mouse and human systems in terms of SAMHD1-related IFN responses might arise from differences in the putative nucleic acid substrates of SAMHD1. There are striking differences between the mouse and human ERVs/LTR elements in terms of their activities, tissue specific expression and temporal regulation during development^35,36^. Given the propensity of these ERVs/LTR elements to affect gene expression, the differences of the repertoire of ERVs/LTR elements could account for the differential susceptibilities of humans and mice to SAMHD1-regulated IFN responses. Another possibility is that an unknown RNA sensor or other components involved in the constitutive IFN-induced signaling pathway could be missing in mice. Recently, a study reported that a cGAS/STING-dependent IFN response is triggered in *Samhd1*-deficient mice, although that study did not address which substrates are involved in the IFN response^37^. Mice contain “active” LTR elements and significant ERV activity^35^. By contrast, “active” ERVs/LTR elements in humans, while present in comparable numbers, have not been described^38^. Thus, the reverse transcribed DNAs originating from the mouse ERVs/LTR elements might be a predominant source of the cGAS/STING-mediated IFN response. Furthermore, these disparities could cause the discrepancy of the source of IFN response between humans and mice, leading to the absence of systemic autoimmune phenotypes in *Samhd1*-deficient mice compared with SAMHD1-related AGS patients.

Notably, SAMHD1 specifically targets retroviral RNA for degradation^15,32^. Even though SAMHD1-CLIP failed to identify dominant substrates and specific binding motifs of SAMHD1, many retroelement RNAs were bound to SAMHD1. Our RNA-seq analysis further displayed that in *SAMHD1*-deficient THP-1 cells, many endogenous retroelement RNAs, primarily LINEs and LTR elements, are upregulated significantly. Therefore, the accumulated retroelement-derived RNA transcripts could be direct substrates of SAMHD1, functioning as immunostimulatory RNAs to activate the type I IFN response. The fundamental question that still remains unanswered is which RNA sensors are involved in linking *SAMHD1*-deficiency to an IFN response. In addition, PI3K/AKT plays an important role in multiple signaling pathway and regulating various cellular function^39^. AKT performs diverse tasks by controlling numerous downstream effectors. Therefore, downstream effector of AKT which directly activate IRF3 needs to be identified. On the other hand, we also observed that lots of genes are significantly regulated by *SAMHD1*-deficiency in direct or indirect manner and various RNA species, which include small nucleolar RNAs (snoRNAs), are bound to SAMHD1 protein. snoRNAs are known to guide the modification (2′-O-methylation and pseudouridylation) of RNAs represented by rRNAs^40^ and the incorporation of 2′-O-methylated nucleosides or pseudouridine into RNA diminishes immunostimulatory activity^41,42^. Therefore, SAMHD1 would indirectly affect the immunostimulatory activity of endogenous RNAs by regulating RNA modification, accounting for a partial role for entire type I IFN response in *SAMHD1*-deficient cells. Considering the phenotypical overlap of AGS with SLE, the pathology of SLE patients, in some cases, might be associated with the accumulation of aberrantly metabolized endogenous nucleic acids. A greater understanding of the pathogenesis of type I interferonopathies is not only of scientific interest but might lead to new therapies for autoinflammatory diseases.

## Methods

### Generation of knockout cell lines and human blood cell isolation

Wild-type and mutants of *SAMHD1* were amplified by PCR from previously described plasmids^15^ and inserted into the EGFP-N3 vector. *SAMHD*1-deficient THP-1, HEK239T, and HeLa cells were generated using the CRISPR/Cas9 system. The *SAMHD1*/*AKT* double knockout THP-1 cell line was established by transfecting *SAMHD1*-deficient THP-1 cells with single guide RNAs (sgRNAs) specific for *AKT1*. Immunoblotting and genomic DNA sequencing confirmed all the knockout cell lines. Guide RNA sequences of *SAMHD1* and *AKT1* are listed in Supplementary Table S1. THP-1 was cultured in Roswell Park Memorial Institute (RPMI) 1640 supplemented with 10% fetal bovine serum (FBS), 2 mM GlutaMAX-I, and 1% penicillin/streptomycin. 293T cells were maintained in Dulbecco’s modified Eagle’s medium (DMEM) supplemented with 10% FBS, 2 mM GlutaMAX-I, and 1% penicillin/streptomycin. PBMCs were isolated from human blood of healthy donors using SepMate, according to the manufacturer’s instruction (Stemcell). Isolated PBMCs were transfected with *SAMHD1*-specific siRNA or control non-specific siRNA for two cycles to obtain efficient knockdown and incubated in RPMI media. After 48 h of incubation, PBMCs were analyzed by western blotting analysis to monitor the activation of STAT1 and AKT.

### Reagents and antibodies

The chemical reagents and antibodies used in this study were purchased from the following manufacturers: poly (I:C) and poly (dA:dT), Sigma; T4 Polynucleotide Kinase and T4 RNA ligase, Takara; T4 RNA Ligase 2 (truncated K227Q) and Antarctic Phosphatase, New England Biolabs (NEB); wortmannin and rapamycin, Sigma; MK-2206, A-674563, and Lapatinib, Selleckchem; Ruxolitinib, Invivogen; mouse monoclonal antibodies to IFNAR1 (Millipore) and SAMHD1 (OriGene); rabbit monoclonal antibodies to IRF3 pho-S386 (Abcam), RIG-I, STING, MyD88, TBK1, TBK1 pho-S172, Stat1 pho-Y701, and GSK-3β (all from Cell Signaling Technology); rabbit polyclonal antibodies to IRF3 (Santa Cruz), SAMHD1 (for immunoprecipitation, Bethyl Lab), GAPDH (Ab Frontier), MAVS, TRIF, IRF7, STAT1, Akt, Akt pho-S473, and GSK-3β pho-S9 (all from Cell Signaling Technology); goat polyclonal antibody to hIL-10Rb (R&D systems).

### RNA interference and transfection

All siRNAs were purchased from Dharmacon as ON-Target plus. THP-1 cells were transfected by electroporation with indicated gene-specific siRNAs or non-specific siRNAs using Neon (Invitrogen). Lysates were collected 72 h after transfection for western blotting and qRT-PCR analysis. For nucleic acid stimulation, PMA-differentiated THP-1 cells were transfected with 5 μg/ml of poly (I:C), poly (dA:dT), or isolated DNA or RNA, using Lipofectamine 2000 transfection reagent (Invitrogen) and undifferentiated THP-1 cells were transfected by electroporation with 10 μg/ml of isolated RNA using Neon. Cells were harvested 4 h post transfection, followed by RNA isolation. For reconstitution of SAMHD1-deficient THP-1, THP-1 cells were transfected by electroporation with expression vectors for wild-type or mutant SAMHD1 using Neon. Isolated PBMCs were transfected using Interferin™ transfection reagent (Polyplus-transfection Inc.), according to the manufacturer’s instructions.

### Genomic DNA and RNA preparation

Genomic DNA was purified using a Blood Mini Kit (Qiagen). Total RNA was isolated using the TRIzol reagent (Invitrogen). Fractionation and extraction of small and large RNAs were performed using the NucleoSpin miRNA system (Macherey-Nagel). Separation and purification of cytoplasmic and nuclear RNAs were carried out using a Cytoplasmic & Nuclear RNA purification kit (Norgen). All the purification steps were processed according to the manufacturers’ instructions.

### *In vitro* nuclease assay by immunoprecipitation

Pelleted THP-1 cells were lysed for 30 min at 4 °C in lysis buffer (25 mM Tris-HCl, pH 7.5, 100 mM KCl, 1 mM DTT, 2 mM EDTA, 0.5 mM PMSF, 0.05% NP-40, RNase inhibitor). After sonication, the lysates were centrifuged for 30 min at 13,000 × *g* at 4 °C. anti-SAMHD1 and anti-rabbit IgG antibodies were incubated with Dynabeads for 1 h at room temperature. The conjugated beads were washed sequentially twice each with buffer A (500 mM NaCl, 10 mM Tris-HCl, pH 7.5, 0.05% NP-40, RNase inhibitor) and buffer B (150 mM NaCl, 10 mM Tris-HCl, pH 7.5, 0.05% NP-40, RNase inhibitor). Cell lysates were incubated with the prepared conjugated Dynabeads for 1 h at 4 °C. The immunoprecipitates were washed five times with buffer B, followed by an *in vitro* nuclease assay. The *in vitro* nuclease assay was performed as described previously^15^. In brief, immunoprecipitated proteins were incubated in 20 µl reaction mixtures containing phosphate-buffered saline (PBS) supplemented with 5 mM MgCl_2_, 2 mM dithiothreitol (DTT) and [γ^−32^P]-labeled RNA substrate (A20) at 37 °C for 30 min. The reactions were stopped by the addition of 20 µl of 2 × RNA loading buffer and then boiled at 95 °C for 5 min. RNA was separated on 8 M urea/15% polyacrylamide gels and analyzed using a BAS-2500 phosphorimager (Fujifilm).

### RNA-seq analysis

CLIP-seq and RNA-seq was performed as previously described, with some modifications^43^. Briefly, THP-1 cells were irradiated by 254 nm UV at total 600 mJ/cm^2^ for RNA-protein cross-liking (Spectroline) for SAMHD1-CLIP. The RNAs bound to SAMHD1 were immunoprecipitated with anti-SAMHD1 conjugated beads. After ligation with 3′ adaptors (5′/rApp/TGGAATTCTCGGGTGCCAAGG/ddC/-3′, Integrated DNA Technologies) using T4 RNA Ligase 2, truncated K227Q (NEB), RNAs were labeled with [γ-^32^P]ATP by T4 polynucleotide kinase (Takara). The RNA-protein complex was separated by SDS-PAGE electrophoresis, then transferred onto nitrocellulose membrane (Whatman). The membrane with RNAs was cut by a razor and RNAs were extracted by phenol/chloroform (Ambion), followed by ethanol precipitation for RNA isolation. 5′ adaptors (5′-GrGrUrUrCrArGrArGrUrUrCrUrArCrArGrUrCrCrGrArCrGrA rUrC-3′, Integrated DNA Technologies) were ligated using T4 RNA ligase (Takara). For RNA-seq, total RNA was extracted from wild-type and SAMHD1-deficient THP-1 cells using the TRIzol reagent (Invitrogen), according to the manufacturer’s instructions. Ribosomal RNA was removed from the total RNA using a Ribo-Zero rRNA removal Kit (Epicentre). After RNA fragmentation by RNA fragmentation reagents (Ambion), RNAs of 30–60 nucleotides were purified and ligated with 3′ and 5′ adaptors using T4 RNA Ligase 2, truncated K227Q, and T4 RNA ligase, respectively. 5′ and 3′ adaptor ligated RNAs were reverse transcribed by using the RNA RT primer (RTP; 5′-GCCTTGGCACCCGAGAATTCCA-3′, Integrated DNA Technologies). PCR was performed to generate libraries for high throughput sequencing with the 5′ end Illumina RNA PCR Primer (RP1) and the 3′ end Illumina RNA PCR Primer with index sequences (Index 1–9). Sequencing was performed on a HiSeq. 2000/2500 (Illumina).

For preprocessing, we removed adapter sequences and low-quality ends from CLIP-seq and RNA- seq reads by using Cutadapt version 1.10 with -m 17–match-read-wildcards -O 10 -e 0.1 -q 30,30 -g AATGATACGGCGACCACCGAGATCTACACGTTCAGAGTTCTACAGTCCGACGATC –a TGGAATTCTCGGGTGCCAAGGAACTCCAGTCAC options. Artifact reads were eliminated by the fastx_artifacts_filter command in FASTX-Toolkit (http://hannonlab.cshl.edu/fastx_toolkit/) version 0.0.13.2. We also discarded reads mapped to human rRNA or tRNA by Bowtie2 version 2.2.7 with -t - k 2–very-sensitive options. Tophat version 2.1.1 with–no-coverage-search–b2-very-sensitive (−b2- score-min L, −0.6, −0.9 only for CLIP-seq due to the high error rate) options was used to align these preprocessed sequencing reads against the human reference genome (GRCh37.p13) downloaded from the Reference Sequence (RefSeq) collection. In this alignment process, we also considered repetitive elements defined by the output of RepeatMasker that was downloadable from RefSeq. Based on these alignments, we calculated the read counts for each genomic position by using in- house software that uniformly divided each multi-mapped read to all of the positions it maps to. As previously described^43^, we applied Fisher’s exact test to detect significant peaks (CLIP-seq enriched regions over RNA-seq). One modification in this study was that we calculated p-values for every genomic position in the whole genome background. Then, the p-values were adjusted by qvalue package in R.

RSEM^44^ version 1.2.30 was used to align sequencing reads against all transcripts of the human reference genome (GRCh37.p13) downloaded from the Reference Sequence (RefSeq) collection and estimate gene-level transcript abundances. DEGs were assessed by limma package in R^45^. We first filtered genes with total read counts less than 12 for 6 samples (i.e., 3 replicates of wild-type and 3 replicates of *SAMHD1* knockout cells). We applied trimmed mean of M values (TMM) normalization to read counts for the estimation of scale factors among samples. Then, the voom transformation was applied to the filtered and normalized counts. After this, the usual limma procedure for differential expression analysis was followed. That is, we estimated the fold changes and standard errors by fitting a linear model for each gene and applied empirical Bayes smoothing to the standard errors. Finally, moderated t-statistics and corresponding p-values were computed. Benjamini-Hochberg method was used to adjust p-values for multiple testing.

### Ethical statement

Human blood samples were anonymously provided by the Blood Center of the Korean Red Cross, Seoul, under the approval of the Institutional Review Board of Korean Red Cross with consent for research use. Experiments involving human blood were approved by the Institutional Review Board at Seoul National University (SNUIRB No. E1512/001–004). All experiments were performed in accordance with Seoul National University guidelines.

### Statistical analysis

Statistical analyses were carried out using GraphPad Prism version 5.0 software. Statistical significance was determined by two-tailed Student’s *t*-tests (*p ≤ 0.05, **p ≤ 0.01, ***p ≤ 0.001, ns: not significant).

### Data availability

The datasets generated during or analyzed during the current study are available from the corresponding author on reasonable request.

## Electronic supplementary material

Supplementary Information
