# Supplementary material for: A central role for PI3K-AKT signaling pathway in linking SAMHD1-deficiency to the type I interferon signature
Source: Sci Rep. 2018 Jan 8;8:84. doi: 10.1038/s41598-017-18308-8 (PMC5758801; doi:10.1038/s41598-017-18308-8)
Supplement: Supplementary file 1 — Supplementary Information [file 41598_2017_18308_MOESM1_ESM.pdf]

**Supplementary Information**

# A central role for PI3K-AKT signaling pathway in linking *SAMHD1*-deficiency to the type I interferon signature

Changhoon Oh[1,2,3], Jeongmin Ryoo[1,2], Kiwon Park[1], Baek Kim[4], Michele B. Daly[4], DongYeon Cho[1,2] & Kwangseog Ahn[1,2]*

[1] School of Biological Sciences, Seoul National University, Seoul 08826, Republic of Korea

[2] Center for RNA Research, Institute for Basic Science (IBS), Seoul 08826, Republic of Korea

[3] Department of the Interdisciplinary Program in Genetic Engineering, Seoul National University, Seoul 08826, Republic of Korea

[4] Department of Pediatrics, Emory University School of Medicine, Atlanta, Georgia 30322, USA

* ksahn@snu.ac.kr

## Supplementary Methods

### Quantitative real-time reverse transcription PCR

Total RNA was subjected to cDNA synthesis using the ReverTra Ace qPCR RT Kit (TOYOBO), according to the manufacturer's instructions. The expression levels of various genes were measured using the iCycler iQ real-time PCR detection system (BioRad) using the TOPreal qPCR PreMIX (Enzynomics). The data were normalized to the expression level of β-actin or GAPDH. The primer sets used are listed in Supplementary Table S1.

### Cell cycle analysis

Wild-type or *SAMHD1*-deficient THP-1 cells were incubated in varying serum concentration. After 48 h, cells were harvested and washed twice with PBS. Then the cells were fixed with 70% cold ethanol overnight at 4°C, followed by washing twice with PBS. The cells were then resuspended with 0.5 ml PBS and 5 µl of 20 mg/ml RNase A and 10 µl of 1 mg/ml PI solution were add, followed by analysis on BD FACSCanto II (BD Biosciences).

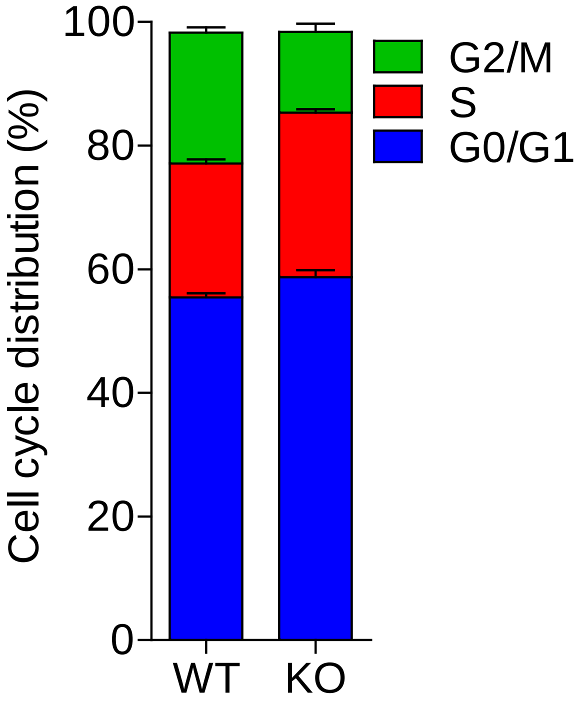


**Supplementary Figure S1. The effect of *SAMHD1*-deficiency on cell cycle distribution.**
Wild-type and *SAMHD1*-deficient cells were synchronized by serum starvation for 24 h, followed by the addition of serum for 24 h to induce cell cycle re-entry. Cells were harvested for propidium iodide (PI) staining and analyzed by FACS to determine the cell cycle distribution. The data represent the mean ± SEM of triplicate independent experiments

A

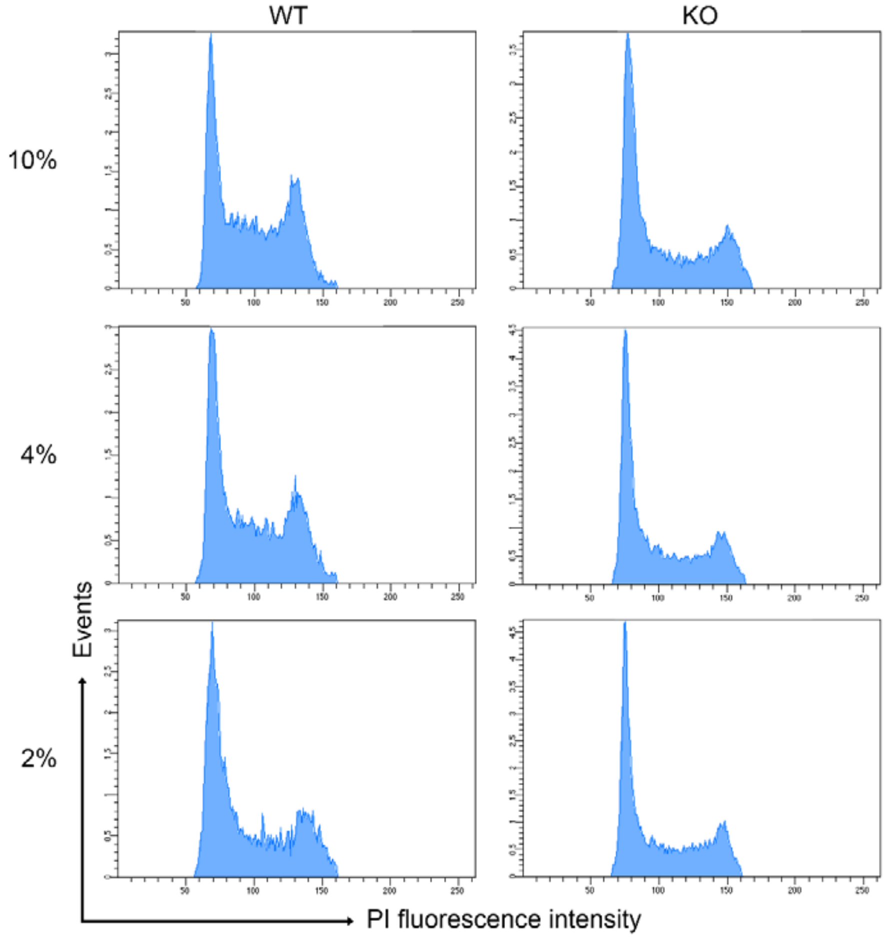


B

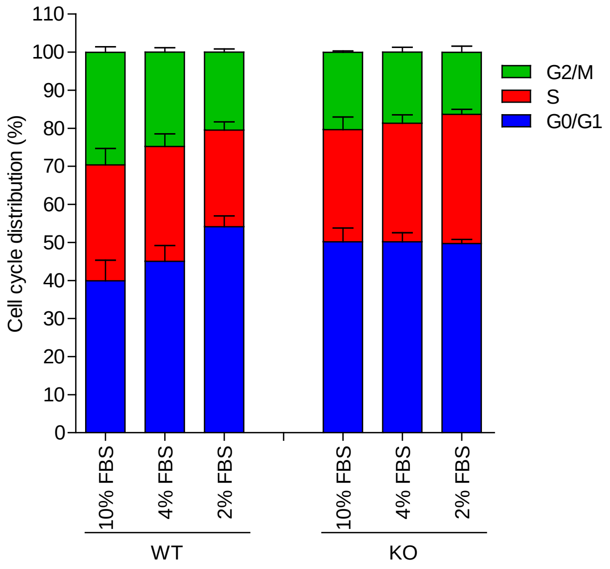


**Supplementary Figure S2. The effect of changes in serum concentration on the manipulation of cell cycle.**
(**A, B**) Wild-type and *SAMHD1*-deficient THP-1 cells were incubated in complete media containing 10 % FBS or reduced-serum media containing 4 % or 2 % FBS for 48 h, followed PI staining and FACS analysis to determine the cell cycle distribution. The data represent the mean ± SEM of triplicate independent experiments.

| Upstream Regulator | Molecule Type | Predicted Activation State | p-value of overlap |
|---|---|---|---|
| IFNA2 | cytokine | Activated | 3.86E-25 |
| IRF3 | transcription regulator | Activated | 4.48E-17 |
| IRF7 | transcription regulator | Activated | 1.4E-15 |
| IRF5 | transcription regulator | Activated | 1.38E-10 |
| STAT1 | transcription regulator | Activated | 8.89E-10 |
| IFNA1/IFNA13 | cytokine | Activated | 2.97E-09 |
| IFNAR1 | transmembrane receptor | Activated | 1.45E-08 |
| IFNB1 | cytokine | Activated | 2.56E-08 |

**Supplementary Figure S3. Bioinformatic prediction of the upstream regulators of DEGs in *SAMHD1*-deficient THP-1 cells.**

Bioinformatic analysis with IPA were performed to identify the upstream regulators of transcriptional regulation. Upstream regulator analysis in IPA sorted out type I IFN and IFN signature associated transcription regulators .

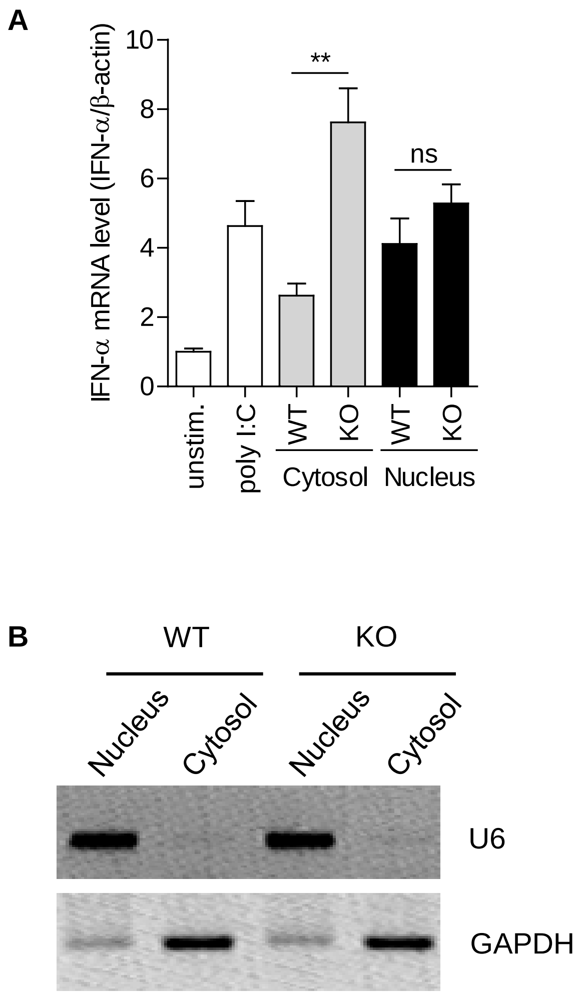


**Supplementary Figure S4. Cytoplasmic RNA from *SAMHD1*-deficient cells activates a type I IFN response.**

(**A**) qRT-PCR analysis of *IFN-α* and *IFN-β* expression in wild-type THP-1 cells stimulated with the nuclear or cytoplasmic RNA purified from wild-type and *SAMHD1*-deficient cells. Data represent the mean ± SEM of triplicate independent experiments (∗∗p ≤ 0.01, ns: not significant, two-tailed Student's t-test). (**B**) RNA recovered from nuclear and cytoplasmic fractions was converted into cDNA. The cDNA was used for PCR amplification of *U6* and *GAPDH*. *U6* and *GAPDH* were used as markers for the quality of the fractionation.

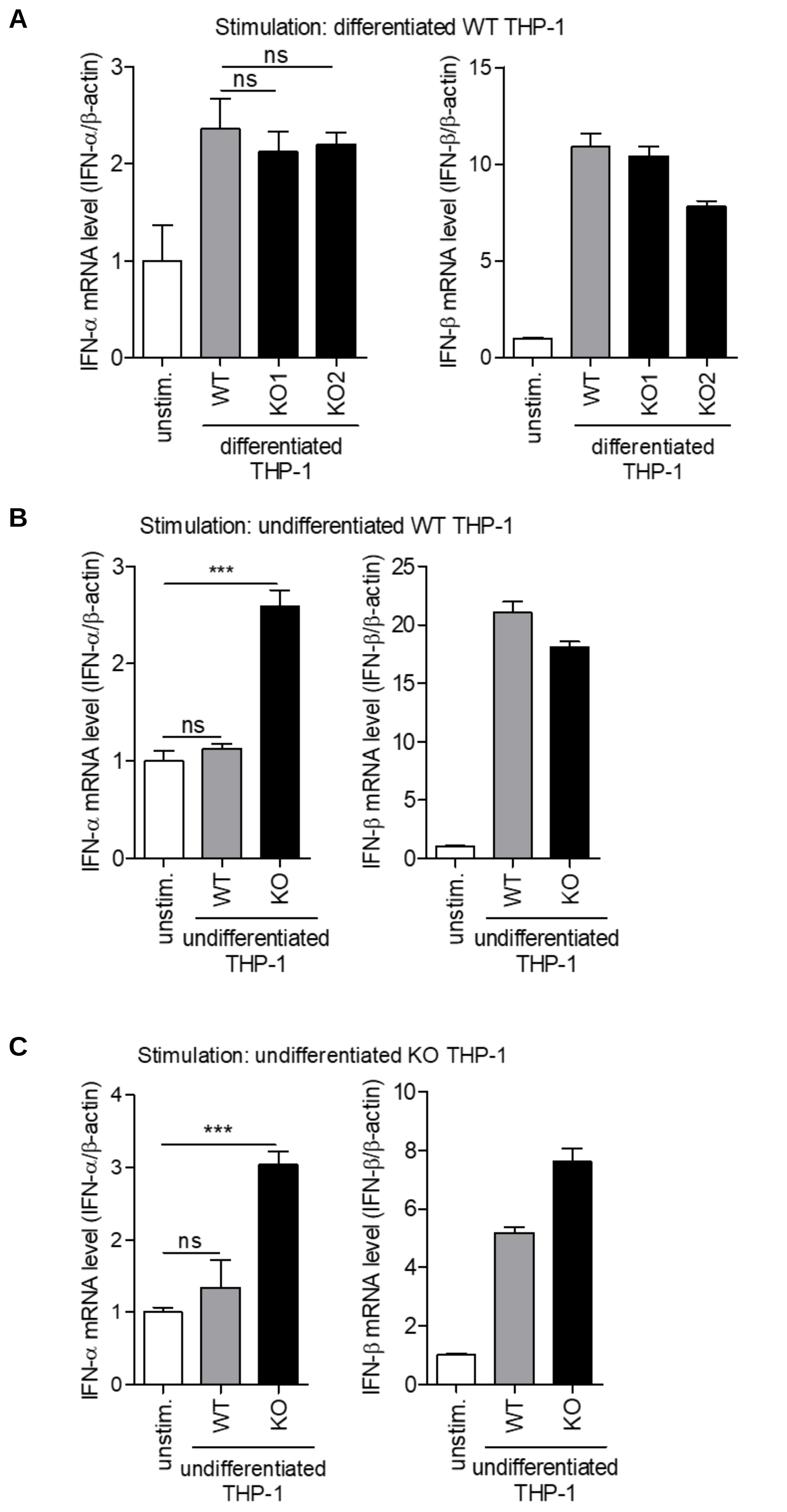


**Supplementary Figure S5. RNA from *SAMHD1*-deficient cells activates a type I IFN response.** (**A**-**C**) PMA-differentiated wild-type THP-1 cells were stimulated with 5 µg/ml of RNA isolated from PMA-differentiated wild-type and *SAMHD1*-deficient cells, or left unstimulated (**A**). Undifferentiated wild-type (**B**) or *SAMHD1*-deficient THP-1 cells (**C**) were stimulated with 10 µg/ml of RNA isolated from undifferentiated wild-type and *SAMHD1*-deficient cells, or left unstimulated. The mRNA levels of *IFN-α* and *IFN-β* were determined by qRT-PCR. Data represent the mean ± SEM of triplicate independent experiments (***$p \leq 0.001$, ns: not significant, two-tailed Student's t-test).

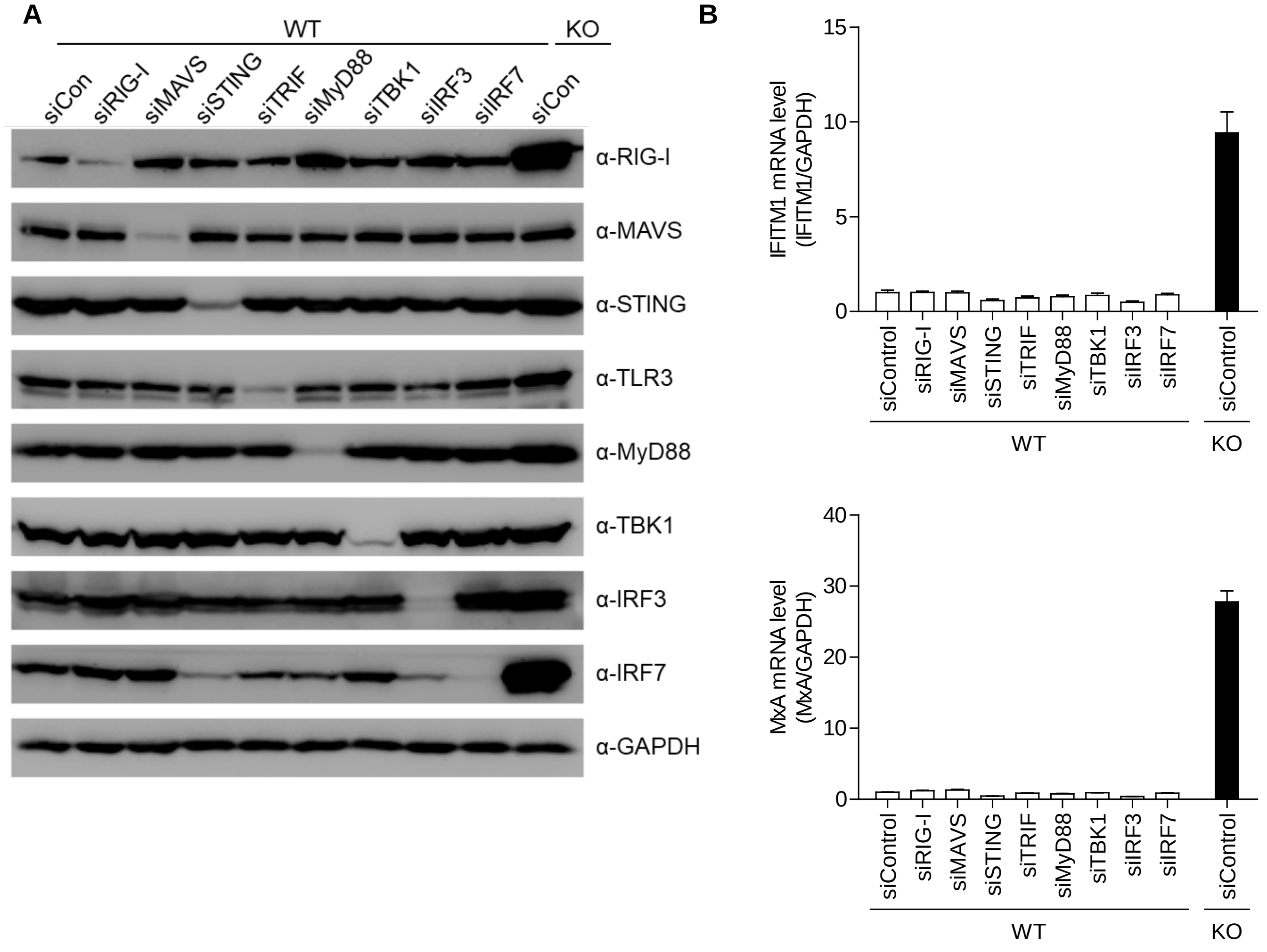


**Supplementary Figure S6. The effect of signaling intermediates knockdown on the activation of type I IFN response.**
(**A, B**) Wild-type and *SAMHD1*-deficient THP-1 cells were transfected with control siRNA or specific siRNA for the indicated genes for 72 h. Cell lysates were subjected to western blotting to analyze the protein levels. GAPDH was loaded as a control (**A**). The cellular mRNA was analyzed by qRT-PCR to determine the expression of *IFITM1* and *MxA* relative to *GAPDH* (**B**). Data represent the mean ± SEM of triplicate independent experiments.

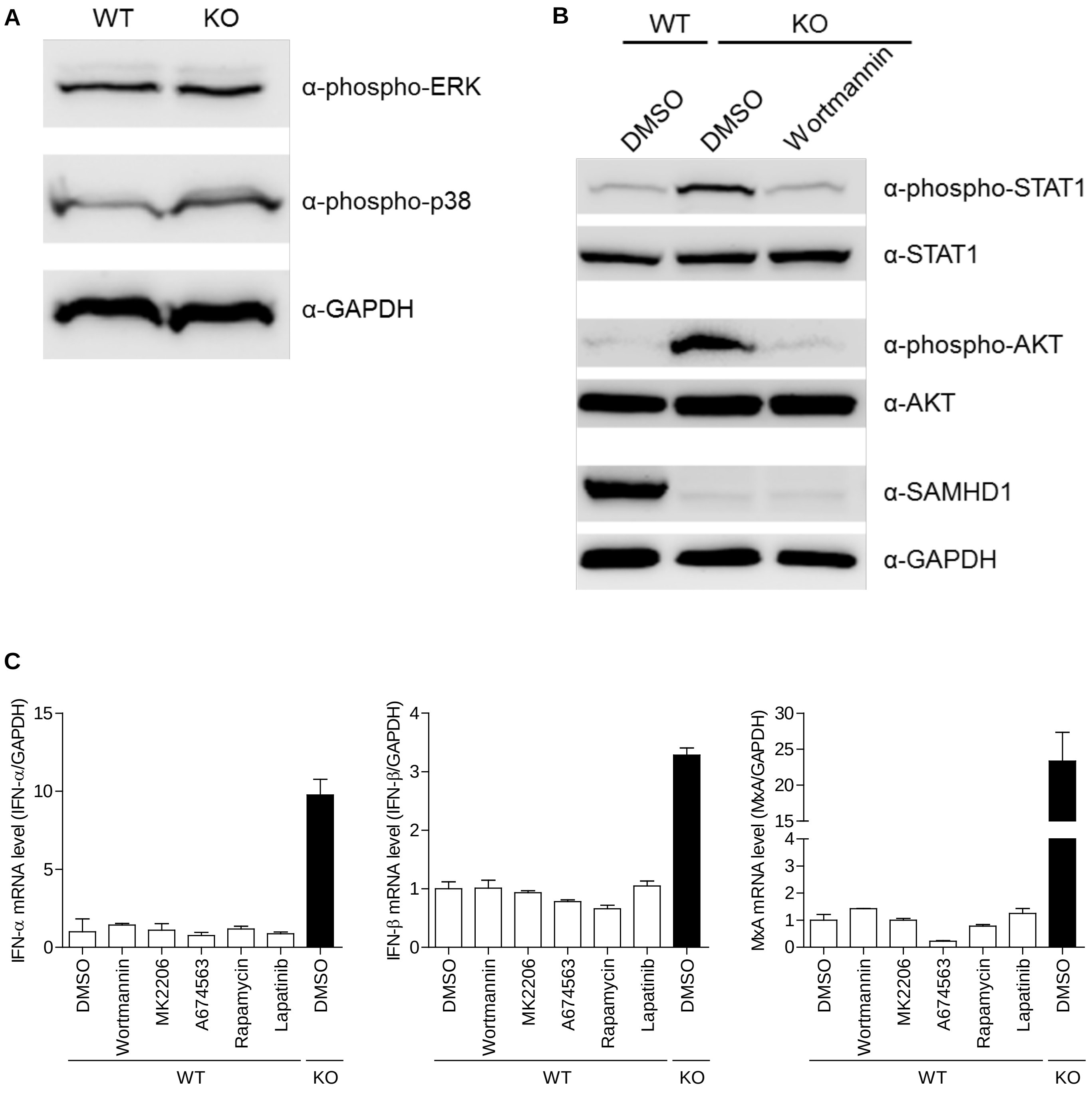


**Supplementary Figure S7. PI3K inhibitor abolishes the activation of STAT1 and AKT.**
(**A**) Western blotting analysis of cell extracts from wild-type and *SAMHD1*-deficient cell lines using the indicated antibodies. (**B**) Wild-type and *SAMHD1*-deficient THP-1 cells were treated with control DMSO or 1 μM Wortmannin for 24 h. Cell lysates were analyzed for the western blotting analysis to determine the phosphorylation status of STAT1 and AKT. GAPDH serves as a loading control. (**C**) Wild-type and *SAMHD1*-deficient THP-1 cells were treated with control DMSO, 1 μM Wortmannin, 1 μM MK2206, 1 μM A674563, 0.1 μM Lapatinib or 50 nM Rapamycin for 24 h. Cells were analyzed for the mRNA levels of *IFN-α*, *IFN-β* and *MxA* relative to *GAPDH* by qRT-PCR. Data represent the mean ± SEM of triplicate independent experiments.

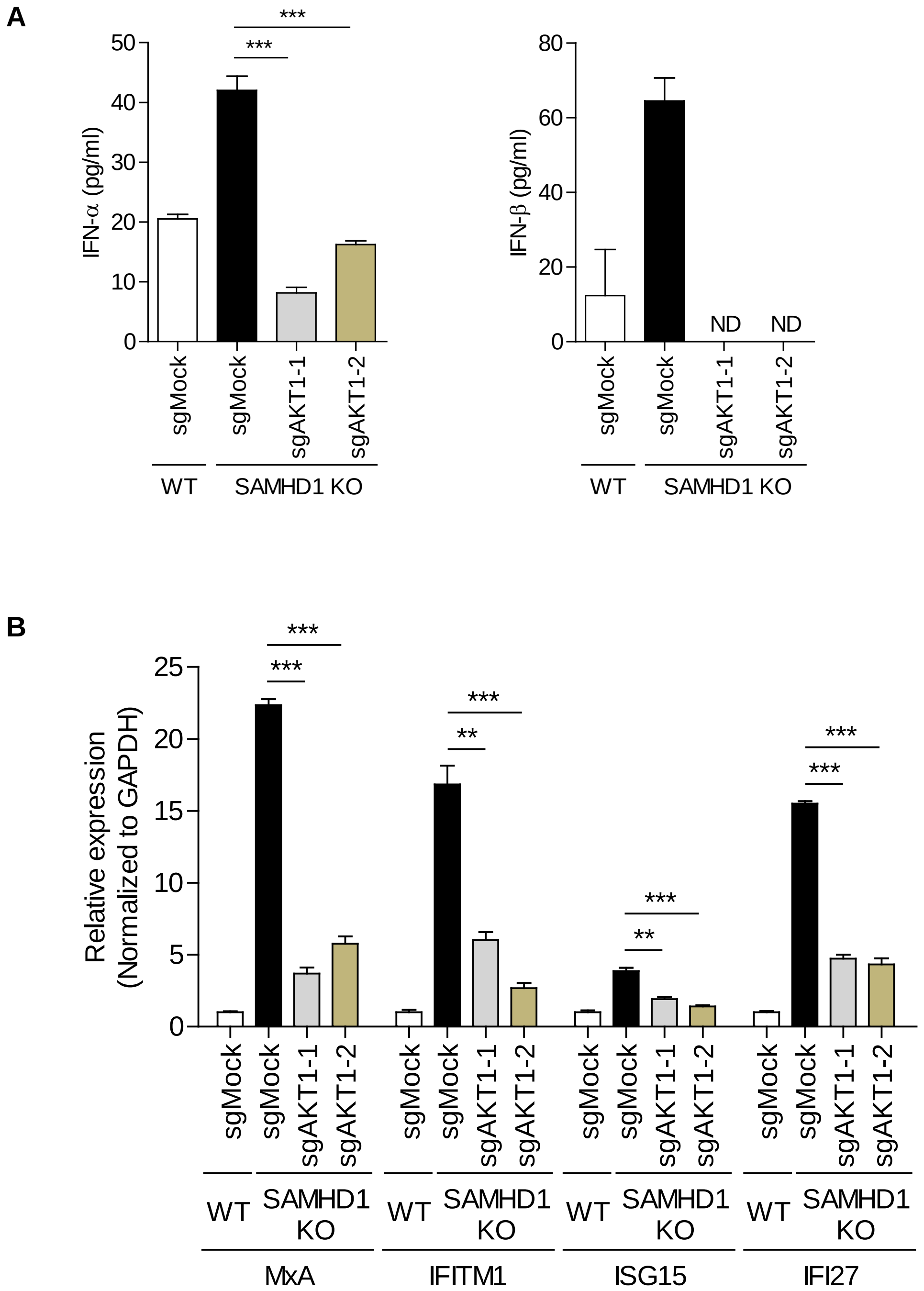


**Supplementary Figure S8. *AKT* knockout abrogates SAMHD1-associated AGS phenotypes.**
(**A, B**) Determination of type I IFN and ISGs production in *SAMHD1*/*AKT1* double knockout THP-1 cells compared with wild-type and *SAMHD1* single knockout cells. Using independent sgRNAs (sgAKT1-1 and sgAKT1-2) targeting *AKT1*, two independent *SAMHD1*/*AKT* double knockout clones with a *SAMHD1*-null background were established and used for the experiments. IFN-α in cell extracts and IFN-β in supernatants were measured using an ELISA. ND, not detectable (**A**). The expression of ISGs (*MxA*, *IFITM1*, *ISG15* and *IFI27*) in the same samples as in (A) was measured by qRT-PCR, normalized to *GAPDH* expression (**B**). Data represent the mean ± SEM of triplicate independent experiments (∗∗p ≤ 0.01, ∗∗∗p ≤ 0.001, two-tailed Student's t-test).

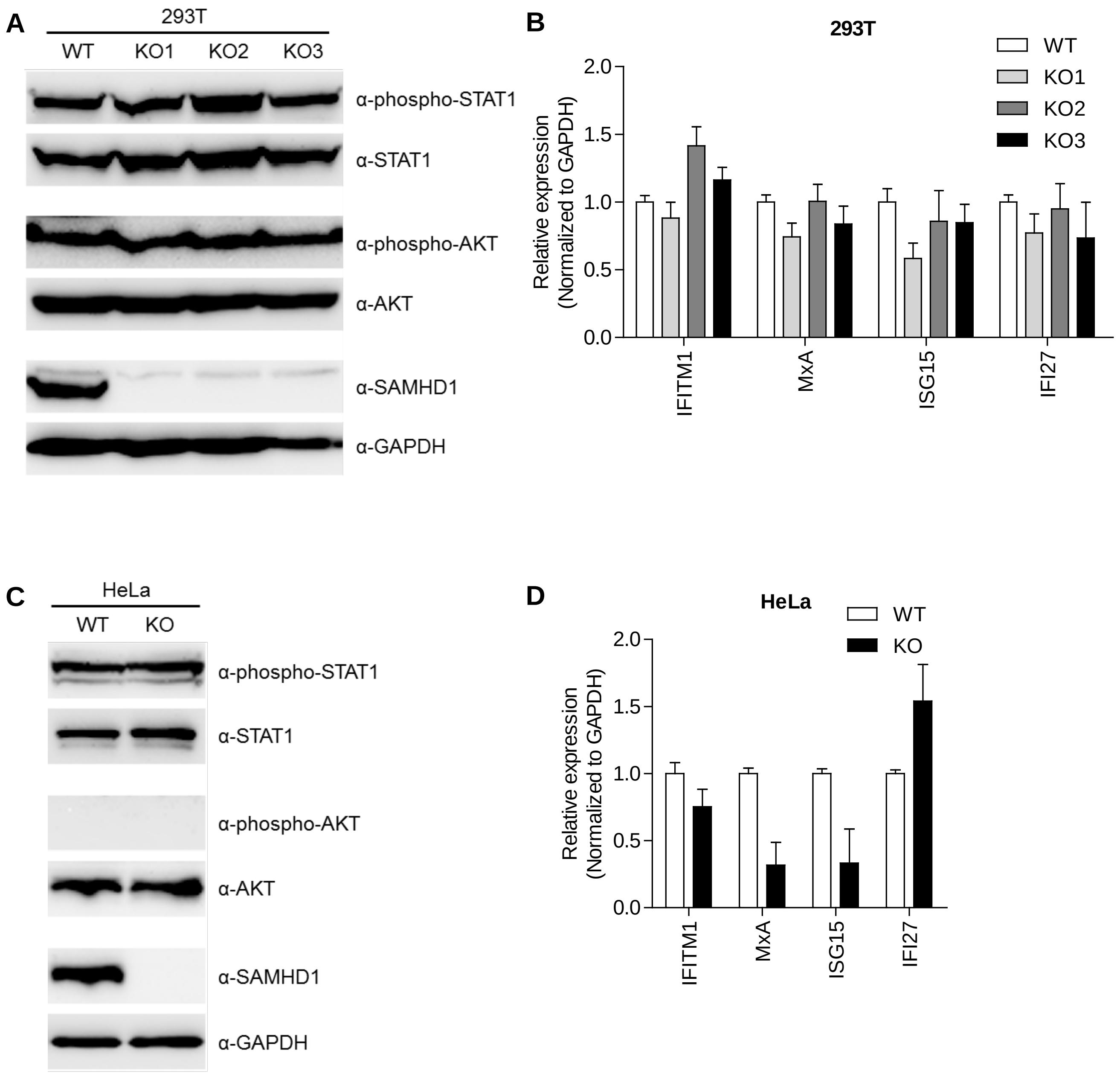


**Supplementary Figure S9. *SAMHD1*-deficient 293T and HeLa cells do not display AGS phenotypes.** (**A-D**) *SAMHD1*-deficient cell lines were constructed in 293T and HeLa cells using the CRISPR/Cas9 system. Western blotting analysis of cell extracts from wild-type and *SAMHD1*-deficient 293T (**A**) and HeLa (**C**) cells. Cell lysates were analyzed for the phosphorylation status of STAT1 and AKT with the indicated antibodies. GAPDH was loaded as a control. The expression of ISGs (*MxA*, *IFITM1*, *ISG15* and *IFI27*) in the same samples as in (A) and (C) were measured by qRT-PCR, respectively, normalized to *GAPDH* expression (**B, D**)

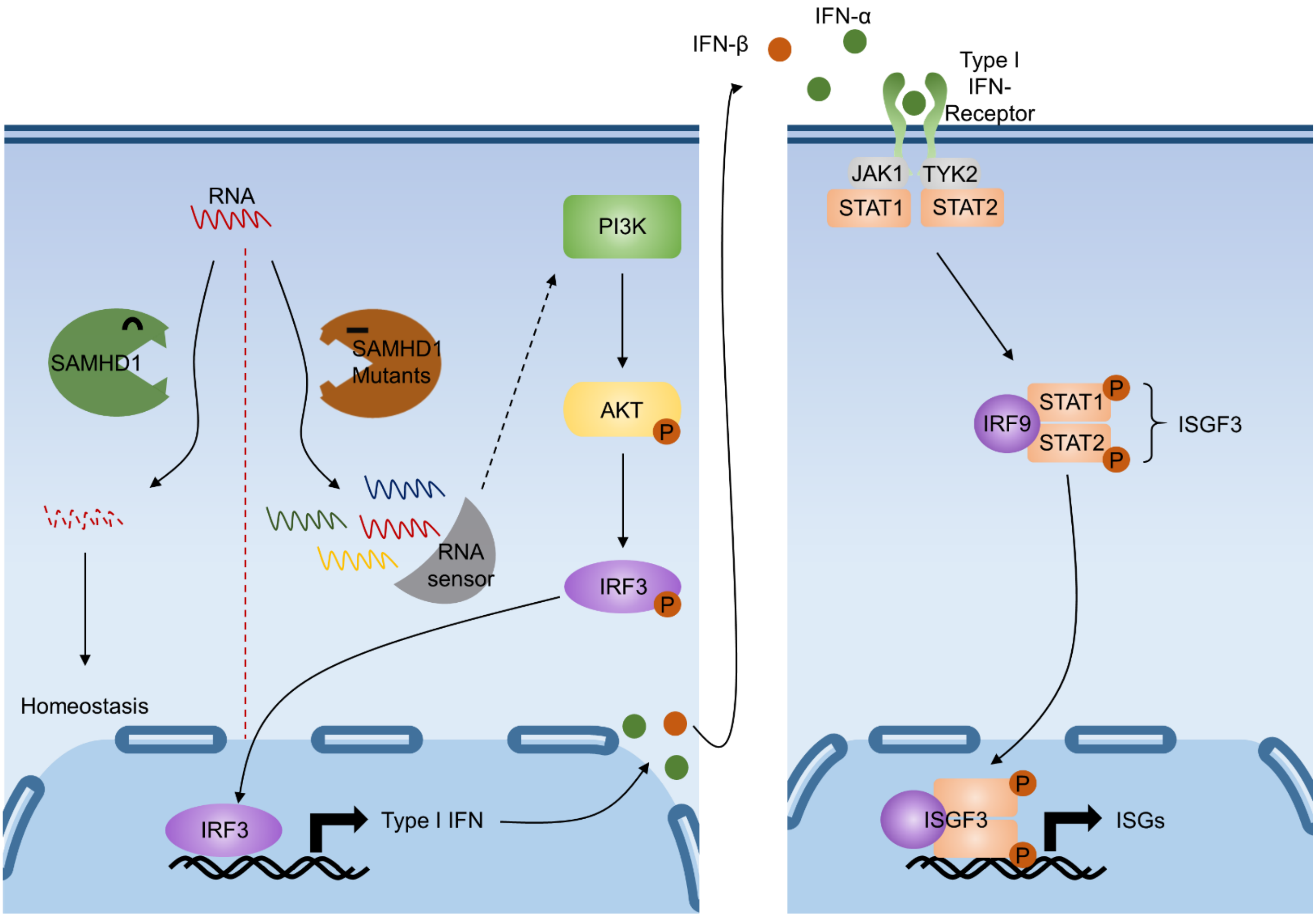


**Supplementary Figure S10. A working model for SAMHD1-related interferonopathy in AGS.** Based on the current study, we propose a working model for linking *SAMHD1*-deficiency to a type I IFN response. In the absence of functional SAMHD1, inappropriately metabolized RNAs accumulate in cells. An unknown RNA sensor recognizes the accumulated RNAs and triggers the downstream activation of the PI3K/AKT signaling pathway, resulting in the expression of type I IFN through IRF3 activation. Finally, binding of type I IFN to the type I IFN receptors of bystander cells activates the JAK/STAT signaling pathway, leading to the induction of ISGs.

| mRNA expression primer sequences | | |
|---|---|---|
| **Gene Product** | **Forward primer** | **Reverse primer** |
| β-actin | CATGTACGTTGCTATCCAGGC | CTCCTTAATGTCACGCACGAT |
| GAPDH | ACCCAGAAGACTGTGGATGG | GGTCCTCAGTGTAGCCCAAG |
| IFNA4 | ACCTGGTTCAACATGGAAATG | ACCAAGCTTCTTCACACTGCT |
| IFNβ | AGTAGGCGACACTGTTCGTG | GCCTCCCATTCAATTGCCAC |
| IFITM1 | CCCCCAGCACCATCCTTC | ACCCCGTTTTTCCTGTATTATCTGT |
| MxA | AGGTCAGTTACCAGGACTAC | ATGGCATTCTGGGCTTTATT |
| ISG15 | ACTCATCTTTGCCAGTACAGGAG | CAGCATCTTCACCGTCAGGTC |
| IFIT1 | GCAGCCAAGTTTTACCGAAG | GCCCTATCTGGTGATGCAGT |
| IFIT2 | CGAACAGCTGAGAATTGCAC | CAAGTTCCAGGTGAAATGGC |
| IFIT3 | AGTCTAGTCACTTGGGGAAAC | ATAAATCTGAGCATCTGAGAGTC |
| IFI27 | GGCAGCCTTGTGGCTACTCT | ATGGAGCCCAGGATGAACTTG |
| IFI44L | GTATAGCATATGTGGCCTTGCTTACT | ATGACCCGGCTTTGAGAAGTC |
| RSAD2 | AGGTTCTGCAAAGTAGAGTTGC | GATCAGGCTTCCATTGCTC |

| sgRNA sequence | |
|---|---|
| **Genomic Target** | **Target site sequence** |
| SAMHD1-1 | CTCAAACACCCCTTCCGCAG |
| SAMHD1-2 | GGCAGACTGGTCCCCGGGCC |
| AKT1-1 | GCAGGATGTGGACCAACGTG |
| AKT1-2 | TGTTGAGGGGAGCCTCACGT |

**Supplementary Table S1**. **Primer sequences used for mRNA expression and sgRNA sequences for the generation of *SAMHD1* and *AKT1* knockout cell lines.**
